# *De novo* HAPLN1 expression hallmarks Wnt-induced stem cell and fibrogenic networks leading to aggressive human hepatocellular carcinomas

**DOI:** 10.18632/oncotarget.9346

**Published:** 2016-05-13

**Authors:** Sihem Mebarki, Romain Désert, Laurent Sulpice, Marie Sicard, Mireille Desille, Frédéric Canal, Hélène Dubois-Pot Schneider, Damien Bergeat, Bruno Turlin, Pascale Bellaud, Elise Lavergne, Rémy Le Guével, Anne Corlu, Christine Perret, Cédric Coulouarn, Bruno Clément, Orlando Musso

**Affiliations:** ^1^ Inserm, UMR991, Liver Metabolisms and Cancer, Rennes, France; ^2^ Université de Rennes 1, Rennes, France; ^3^ Inserm, Institut Cochin, Paris, France; ^4^ Cnrs, UMR8104, Paris, France; ^5^ Université Paris Descartes, Sorbonne Paris Cité, Paris, France; ^6^ CHU de Rennes, Centre de Ressources Biologiques Santé, Rennes, France; ^7^ Université de Rennes 1, UMS 18 Biosit, Biogenouest, Rennes, France; ^8^ CHU de Rennes, Department of Gastrointestinal and Hepatobiliary Surgery, Rennes, France; ^9^ Université de Rennes 1, UMS 18 Biosit, Biogenouest, ImPACcellCore Facility, Rennes, France

**Keywords:** Wnt, β-catenin, epithelial-mesenchymal transition, LGR5, CD44, Gerotarget

## Abstract

About 20% hepatocellular carcinomas (HCCs) display wild-type β-catenin, enhanced Wnt signaling, hepatocyte dedifferentiation and bad outcome, suggesting a specific impact of Wnt signals on HCC stem/progenitor cells. To study Wnt-specific molecular pathways, cell fates and clinical outcome, we fine-tuned Wnt/β-catenin signaling in liver progenitor cells, using the prototypical Wnt ligand Wnt3a. Cell biology assays and transcriptomic profiling were performed in HepaRG hepatic progenitors exposed to Wnt3a after β-catenin knockdown or Wnt inhibition with FZD8_CRD. Gene expression network, molecular pathology and survival analyses were performed on HCCs and matching non-tumor livers from 70 patients by real-time PCR and tissue micro-array-based immunohistochemistry. Wnt3a reprogrammed liver progenitors to replicating fibrogenic myofibroblast-like cells displaying stem and invasive features. Invasion was inhibited by 30 nM FZD7 and FZD8 CRDs. Translation of these data to human HCCs revealed two tight gene networks associating cell surface Wnt signaling, stem/progenitor markers and mesenchymal commitment. Both networks were linked by *Hyaluronan And Proteoglycan Link Protein 1* (HAPLN1), that appeared *de novo* in aggressive HCCs expressing cytoplasmic β-catenin and stem cell markers. HAPLN1 was independently associated with bad overall and disease-free outcome. *In vitro,* HAPLN1 was expressed *de novo* in EPCAM^−^/NCAM+ mesoderm-committed progenitors, upon spontaneous epithelial-mesenchymal transition and de-differentiation of hepatocyte-like cells to liver progenitors. In these cells, HAPLN1 knockdown downregulated key markers of mesenchymal cells, such as Snail, LGR5, collagen IV and α-SMA. In conclusion, HAPLN1 reflects a signaling network leading to stemness, mesenchymal commitment and HCC progression.

## INTRODUCTION

Approximately 20% of HCCs show activation of Wnt/β-catenin and TGFβ pathways, carry wild-type β-catenin, lose the hepatocyte phenotype and overexpress Frizzled (FZD) receptors [1, 2]. Wnt/β-catenin pathway activation involves interaction of Wnt ligands with cell surface FZD receptors and LRP5/6 co-receptors, followed by disruption of the *Adenomatous polyposis coli* (APC)-axin platform, thus halting GSK3B-dependent phosphorylation of β-catenin at key residues in exon 3. Non-phosphorylated β-catenin is thus stabilized, accumulates in the cytoplasm and nucleus and interacts with T-cell factor (TCF) transcription factors of target gene expression. Conversely, in the absence of interaction of Wnt ligands with their cell surface receptors, phosphorylated β-catenin undergoes proteasomal degradation. β-catenin exon 3 mutations in tumors bypass the GSK3B gatekeeper, thus switching the pathway to the *ON* position constitutively [3].

It is widely accepted that Wnt pathway activation in HCCs may be driven by upregulation of TGFB and tyrosine-kinase receptor pathways [2, 4, 5], Wnt ligands, their cell surface frizzled receptors and/or epigenetic silencing of a family of endogenous Wnt inhibitors, i.e., the Secreted Frizzled-Related Proteins (SFRPs) [6]. SFRPs are soluble decoy receptors composed of a *c*ysteine-*r*ich *f*ri*z*zle*d* ligand-binding *d*omain (FZD_CRD), thereby quenching Wnt activity at the cell surface [7]. Thus, increasing the ratio of SFRP-like Wnt inhibitors over Wnt ligands or receptors controls tumor growth [8–11].

Most cases of HCC arise in a background of chronic inflammation and tissue remodeling, leading to stem/progenitor cell amplification [12]. In the context of experimental liver regeneration in response to tissue damage, Wnt signals expand transit-amplifying liver progenitor cells but impair hepatocyte differentiation [13]. Thus, the aim of the present study was to investigate Wnt-specific molecular pathways, cell fates, phenotypes and clinical outcomes in HCC stem/progenitor cells. To this end, we fine-tuned Wnt/β-catenin signaling with the prototypical Wnt3a ligand [14, 15] and inhibited Wnt signaling using β-catenin siRNA knockdown or soluble FZD8_CRD in HepaRG liver progenitor cells. These bipotent progenitors carry wild-type Wnt/β-catenin pathway components [16] and can differentiate into hepatocyte- or biliary-like cells [17, 18].

We report that Wnt3a reprograms liver progenitors to replicating and invasive fibrogenic myofibroblast-like cells expressing stem cell markers. Analysis of HCCs from 70 patients revealed two tight gene networks associating stem/progenitor markers, cell surface Wnt signaling and fibrogenesis. Both networks were linked by *Hyaluronan And Proteoglycan Link Protein 1* (HAPLN1), a mesenchymal matrix protein, which is essential in development [19]. HAPLN1 links proteoglycans with hyaluronic acid, thereby building growth factor binding platforms [20]. Whereas HAPLN1 appeared *de novo* in aggressive tumors expressing stem cell markers and *in vitro* models of epithelial-mesenchymal transition; HAPLN1 knockdown downregulated key markers of mesenchymal cells. We hypothesize that HAPLN1 can be hijacked by tumor evolution as a selective advantage for cancer progression.

## RESULTS

### Wnt signals lead to myofibroblastic differentiation of liver progenitor cells

**Figure 1:**
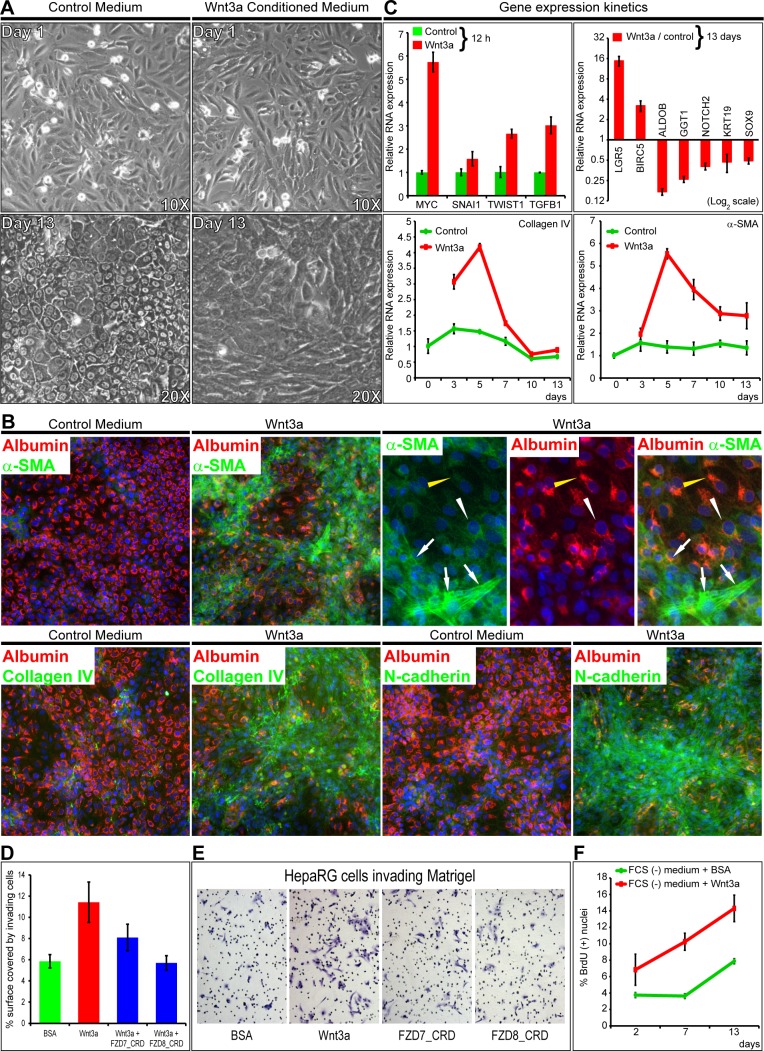
Wnt signals differentiate liver progenitors to myofibroblast-like cells invading Matrigel **A**. and **B**. Incubation of progenitor HepaRG cells with control or Wnt3a-conditioned medium for 13 days. **A**. Controls are hepatocyte-like. Cells incubated with Wnt3a are fibroblast-like. **B**. Coimmunodetection of the indicated proteins. Alpha smooth muscle actin (α-SMA), collagen IV and N-cadherin: green (FITC); albumin, red (FluoProbes 594); nuclei, blue (DAPI). Images were acquired with a Cellomics station at 20X. Wnt3a-incubated cells show bridging fascicules of spindle α-SMA(+)/albumin(−) cells. Stress fibers typical of myofibroblasts *(white arrows)* embrace foci of transitional elongated α-SMA(+)/albumin(+) cells *(white arrowheads)*, with granular and filamentous compartments *(yellow arrowheads)*. **C**. Gene expression kinetics of HepaRG cells incubated with recombinant Wnt3a or control BSA and analyzed by real-time PCR. **D**. FZD7_CRD and FZD8_CRD inhibit Wnt3a-induced Matrigel invasion. Cells were incubated in Matrigel-coated Boyden chambers with FCS(−) medium plus BSA; 7 nM Wnt3a; Wnt3a plus 30 nM FZD7 or FZD8_CRD. **E**. HepaRG cells invading Matrigel. **F**. Wnt3a enhances cell proliferation. Cells were incubated in 96-well plates with FCS(−) medium plus BSA or Wnt3a. BrdU(+) nuclei were counted with a Cellomics station.

As Wnt3a binds frizzled (FZD) receptors at the low nanomolar range [3], HepaRG hepatic progenitor cells were incubated with Wnt3a-conditioned medium, which contains <10 nM Wnt3a [9] or with 7 nM purified recombinant Wnt3a for 13 days (Figure 1A and Supplementary Figure 1A and 1B). Control media led differentiation to hepatocyte-like cells, as expected [18]. In contrast, Wnt3a-enriched media led to albumin^−^ fibroblast-like cells, containing alpha-smooth-muscle-actin+ (α-SMA^+^) stress fibers, thus indicating myofibroblast differentiation (Figure 1B). These cells expressed the basement membrane collagen type IV, the mesenchymal markers N-cadherin (Figure 1B), Vimentin and Fibronectin, as well as cytoplasmic β-catenin (Supplementary Figure 1C). Gene expression kinetics showed increased MYC, SNAI1, TWIST1 and TGFB1 after 12 h, with a peak in Collagen IV and α-SMA mRNAs after 5 days of incubation of progenitor cells with purified Wnt3a. After 13 days, myofibroblast-like cells showed an important increase in LGR5 and BIRC5 (Survivin) and decrease in Aldolase B, GGT1, NOTCH2, Keratin 19 and SOX9 (Figure 1C). Consistently, after Wnt3a treatment, SNAI1 showed a seven-fold increase within the 1^st^ h and CD44 increased within the first 8 h (Supplementary Figure 1D). Wnt3a promoted Matrigel invasion, [inhibited by 30 nM FZD7_CRD or FZD8_CRD (Figure 1D and 1E)], and cell proliferation throughout the 13-day assay (Figure 1F).

### Wnt signals promote commitment of transit-amplifying liver progenitor cells to fibrogenic myofibroblast-like cells

To look at the gene networks leading to myofibroblastic differentiation of hepatic progenitors, we treated cells with 7 nM Wnt3a for three days. To identify β-catenin-dependent or FZD8_CRD sensitive gene networks, we transfected cells with β-catenin-targeting siRNA or treated them with the prototypical Wnt inhibitor FZD8_CRD. This strategy was validated by real-time PCR gene expression analysis of the β-catenin target gene LGR5 in HepaRG and Huh7 cells (Supplementary Figure 2A-2D). A whole transcriptome analysis identified 358 genes differentially regulated by Wnt3a in progenitor HepaRG cells (Supplementary Figure 2E-2H and Supplementary Table 3A). After filtering out 169/358 genes affected by control (scrambled) siRNA (Supplementary Table 3B), we obtained 189 genes, 142 (75%) of them were sensitive to β-catenin siRNA knockdown and 47 (25%) were not (Supplementary Table 3, C and D). Among the 358 genes modulated by Wnt3a in HepaRG cells, 233 (65%) of them were inhibited by 30 nM FZD8_CRD and 125 (35%) were not (Supplementary Table 3, E and F). Finally, 77 genes were both sensitive to both β-catenin knockdown and FZD8_CRD in independent experiments (Supplementary Table 3G).

**Figure 2:**
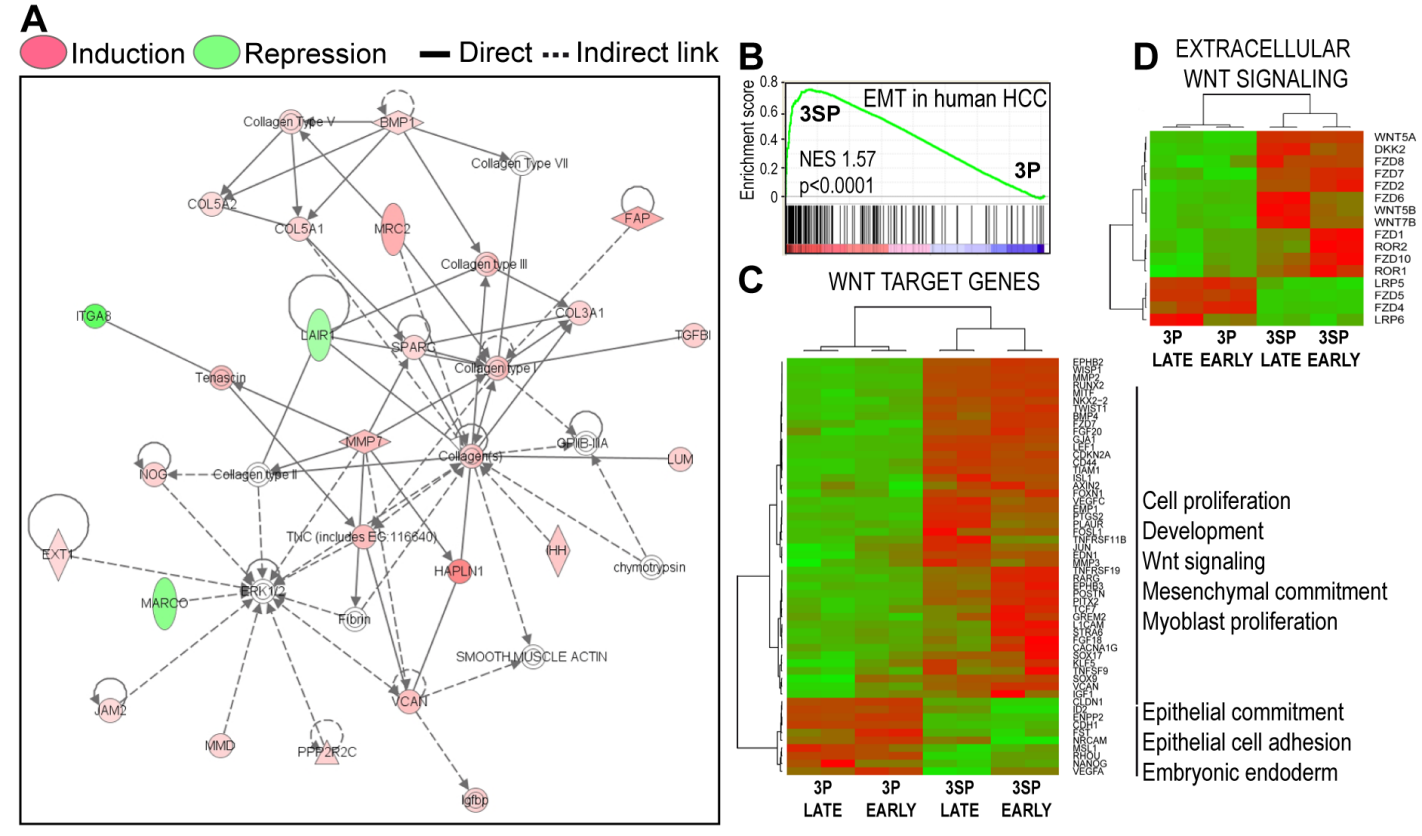
Wnt signals activate a fibrogenic gene expression program in liver progenitors **A**. Gene networks applying Ingenuity pathway analysis on the 358 gene set differentially regulated by Wnt3a. **B**. Wnt-induced differentiation of hepatic progenitors into fibrogenic myofibroblast-like cells matches the transcriptome signature of spontaneously emerging EMT in human HCC (GSE26391). 3P and 3SP human HCC cell lines derive from the same tumor, mesenchymal 3SP resulting from epithelial 3P cells that underwent spontaneous EMT in a human HCC [23]. By gene set enrichment analysis, 3SP cells are significantly enriched in transcripts induced by Wnt3a in HepaRG cells. **C**. and **D**. Hierarchical clustering: 3SP and 3P cells are distinguished by C *Wnt target genes* from the Wnt Homepage (Supplementary Table 6A) and D *Extracellular Wnt signaling* (Wnt pathway ligands and receptors). Raw gene expression data were normalized by the Robust Multi-array Average (RMA) log_2_ algorithm and quantile filtered.

Ingenuity Pathway Analysis revealed fibrogenesis and myofibroblast activation pathways (Figure 2A). Consistently, gene ontology analysis by FuncAssociate 2.0 [21] confirmed that Wnt signals induced highly significant and reproducible fibrogenic gene sets (Supplementary Table 4). Also, gene ontology analysis confirmed that the gene set affected by scrambled siRNA was only associated with actin crosslink formation, reflecting cytoskeletal rearrangements following electroporation [22]. Importantly, genes specifically silenced by β-catenin targeting siRNA and by FZD8_CRD were both associated with fibrogenesis.

To strengthen our observations, we compared the Wnt-induced signature in HepaRG cells with the whole genome mRNA expression profiles of epithelial 3P and fibroblast-like 3SP human HCC cell lines. These cells are derived from the same HCC, 3SP resulting from 3P cells that underwent spontaneous EMT [23]. Gene Set Enrichment Analysis (GSEA) revealed that the transcriptome of 3SP cells was significantly enriched in genes induced by Wnt3a in HepaRG progenitor cells (Figure 2B). By hierarchical clustering, fibroblast-like 3SP cells were unambiguously distinguished from epithelial 3P cells on the basis of the gene expression of Wnt Homepage targets (Figure 2C) and extracellular Wnt pathway components (Figure 2D) and associated functions (Supplementary Table 5). Moreover, comparison of the mRNA signature of transiently mesenchymal-competent cells induced by dedifferentiation of hepatocyte-like HepaRG cells [18] with our Wnt3a-induced signature confirmed that Wnt signals specifically promote differentiation of transit-amplifying liver progenitor cells to fibrogenic myofibroblast-like cells (Supplementary Table 6).

### *In vitro* Wnt-induced fibrogenesis activates signaling networks that are functional in HCCs

**Figure 3:**
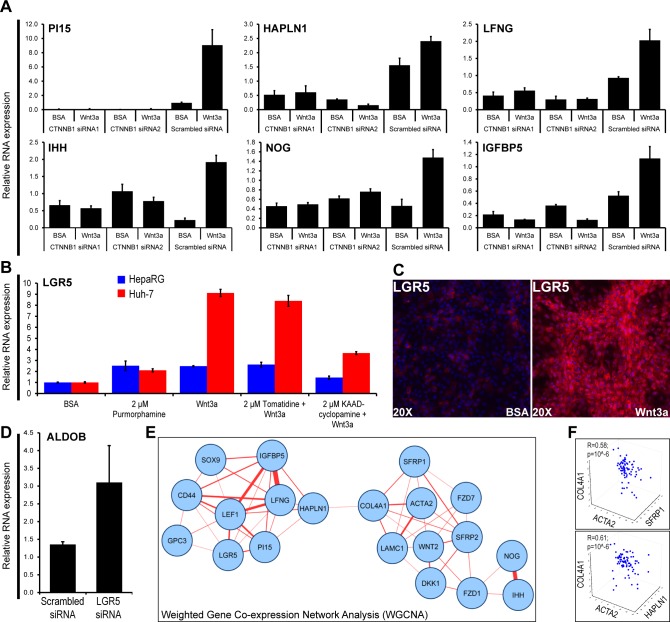
*In vitro* Wnt-induced fibrogenesis activates signaling networks that are functional in HCCs **A**. RNA levels of the indicated genes in progenitor HepaRG HCC cells treated with Wnt3a after transfection with two β-catenin targeting siRNAs. **B**. LGR5 mRNA expression in HepaRG and Huh-7 cells treated with BSA (control), the Hedgehog pathway inducer Purmorphamine, Wnt3a and the Hedgehog inhibitor KAAD-cyclopamine. Tomatidine is a cyclopamine structural analog which does not inhibit the Hedgehog pathway. **C**. Immunodetection of LGR5 in HepaRG cells after 13 days of incubation with Wnt3a or BSA. LGR5, red (FluoProbes 594); nuclei, blue (DAPI). **D**. RNA expression of the hepatocyte differentiation marker ALDOB in HepaRG cells transfected with LGR5 siRNA. **E**. Gene networks generated by Weighted Correlation Network Analysis (WGCNA) [44] using mRNA expression data for the indicated genes in 79 HCCs. Networks with a correlation coefficient threshold ≥0.30 plotted with Cytoscape [45]. Closeness between nodes is proportional to the number of connections. Thickness of links is proportional to the correlation coefficients. **F**. 3D scatterplots showing the relationships between mRNA expression of the indicated genes in 79 HCCs. ACTA2, smooth muscle actin; COL4A1, procollagen, type IV, alpha 1 chain. Multiple regression coefficients *(R)* are indicated.

Close analysis of Wnt-induced genes sensitive to both β-catenin targeting siRNA and to FZD8_CRD (Supplementary Table 3G) revealed crosstalks of Wnt with TGFB/BMP, Notch, Hedgehog and IGF signaling pathways [5]. To validate these crosstalks, β-catenin dependency of representative genes of these four signaling pathways was confirmed by real-time PCR (Figure 3A). Among these, the induction of the stem cell marker LGR5 by Wnt3a in Huh-7 or HepaRG cells was partially inhibited by the Hedgehog inhibitor KAAD-cyclopamine, but not by the inactive structural analog tomatidine (Figure 3B). Whereas LGR5 expression by myofibroblast-like cells suggested that they retained stem cell features (Figure 3C); silencing of LGR5 by specific siRNA upregulated the hepatocyte differentiation marker aldolase B (Figure 3D), suggesting that Wnt-induced LGR5 impairs hepatocyte differentiation.

We then sought to validate these findings in 79 HCCs, by assessing the mRNA expression of the genes shown in Figure 3A-3C, together with genes involved in cell surface Wnt signaling (GPC3, SFRP1, SFRP2, DKK1, FZD1, FZD7, WNT2) and mesenchymal commitment (ACTA2, LAMC1; COL4A1). Of note, SFRPs 1 and 2 also reflect mesenchymal commitment of stem cells [24]. Weighted Gene Co-expression Network Analysis revealed two tight networks associating stem/progenitor markers to cell surface Wnt signaling and mesenchymal commitment (Figure 3E and Supplementary Table 7). Both networks were linked by HAPLN1 and collagen IV (COL4A1). *Hyaluronan And Proteoglycan Link Protein 1* (HAPLN1) is a cartilage gene that links proteoglycans to hyaluronic acid, thereby playing a key role in extracellular matrix assembly [20]. While its absence leads to dwarfism [19], tumors *de novo* expresses HAPLN1 [25]. Multiple correlation analyses confirmed that both SFRP1 and HAPLN1 were associated with ACTA2 (α-SMA) and collagen type IV (Figure 3F).

### *De novo* expression of HAPLN1 hallmarks mesenchymal commitment

**Figure 4:**
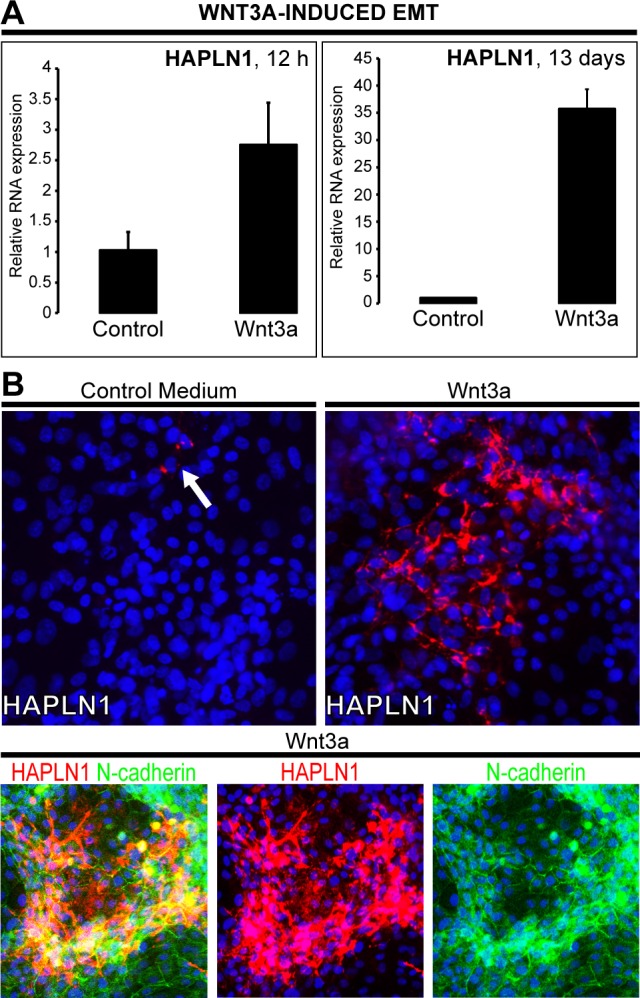
HAPLN1 upregulation by Wnt3a over 13 days HepaRG progenitor cells were cultured with control FCS(−)/BSA(+) or FCS(−)/BSA(+)/Wnt3a. **A**. HAPLN1 mRNA levels 12 h and 13 days upon Wnt3a-induction. **B**. Immunodetection of HAPLN1, red (Cy5); N-cadherin, green (FITC). Nuclei, blue (DAPI). Images acquired at 20X. Control (BSA) shows very rare cells expressing HAPLN1 *(arrow).* HAPLN1 labels the pericellular matrix and colocalizes with N-cadherin.

**Figure 5:**
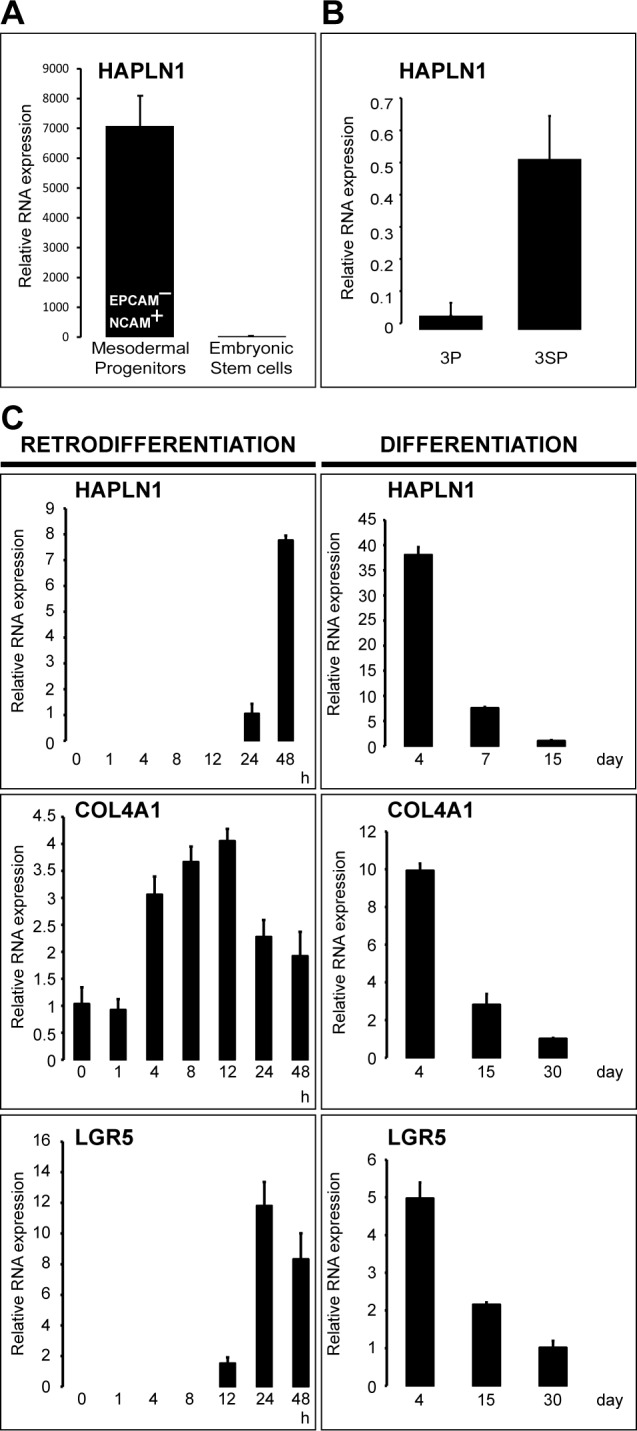
HAPLN1 hallmarks early mesoderm commitment of pluripotent hES cells and spontaneous EMT **A**. Normalized mRNA data from GSE21668 [26] showing *de novo* HAPLN1 expression in early EPCAM−/NCAM+ mesoderm-committed progenitors generated from undifferentiated human embryonic stem cells. **B**. HAPLN1 expression analyzed by real-time PCR in the 3P→3SP human HCC cell lines derived from the same tumor. Mesenchymal 3SP result from epithelial 3P cells that underwent spontaneous EMT [23]. **C**. Kinetics of mRNA expression analyzed by real-time PCR in HepaRG cells along the retrodifferentiation of hepatocytes into liver progenitors and the differentiation of liver progenitors to hepatocytes. Time points 0 h and 30 days (COL4A1 and LGR5) or 15 days (HAPLN1) were used as calibrators. HAPLN1 was not detectable at 30 days in differentiated hepatocyte-like cells.

**Figure 6:**
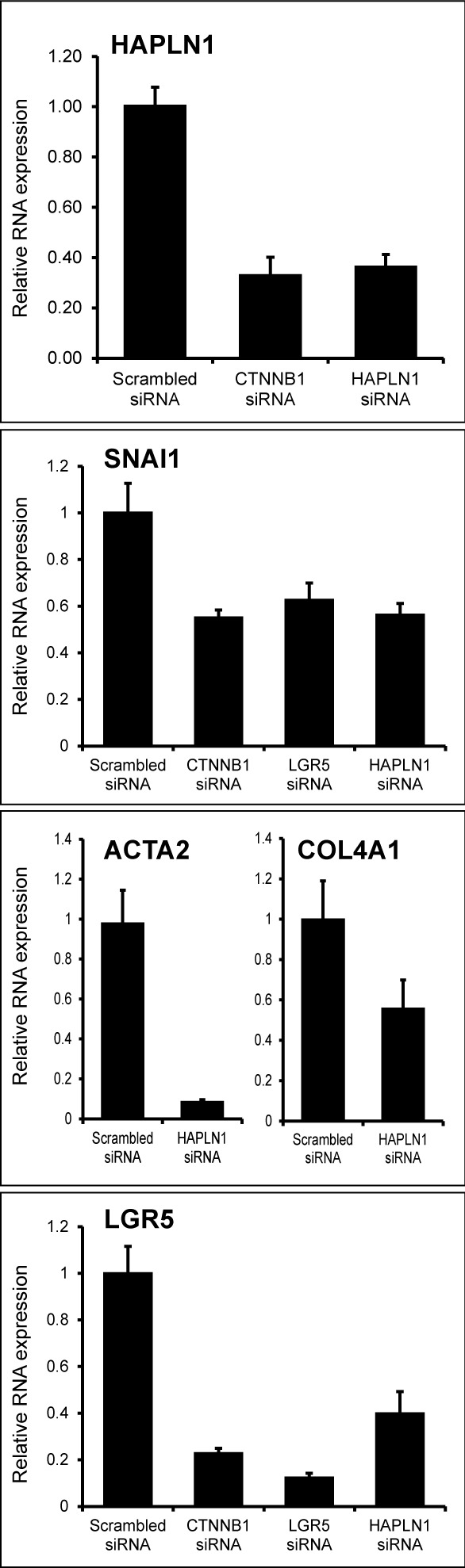
HAPLN1 silencing leads to mesenchymal and stem cell marker loss Real-time-PCR-assessed mRNA expression of the indicated genes in HepaRG progenitor cells transfected with the indicated siRNAs. HAPLN1 silencing downregulates SNAI1; ACTA2; COL4A1 and LGR5 expression.

Wnt3a-induced upregulation of HAPLN1 was first detected at 12 h (~2.5 fold) reaching >30 folds at 13 days. At this time, HAPLN1 was deposited in the pericellular matrix of N-cadherin^+^ cells (Figure 4A and 4B). Generation of EPCAM^−^/NCAM^+^ cells is the earliest stage of mesoderm commitment from undifferentiated human embryonic stem cells [26]. Analysis of this dataset (GSE21668) revealed *de novo* HAPLN1 expression at the earliest stages of mesoderm commitment (Figure 5A). Consistently, by real-time PCR, fibroblast-like HCC 3SP cells showed higher HAPLN1 mRNA levels than epithelial 3P cells (Figure 5B). Moreover, kinetics analysis of gene expression, upon dedifferentiation of hepatocyte-like HepaRG cells to transient mesenchyme-competent progenitors, revealed that HAPLN1 became detectable at 24 h and increased by 8 folds at 48 h post plating. Conversely, upon differentiation of these progenitors to hepatocyte-like cells, HAPLN1 decreased by >35 folds from day 4 to day 15 and was no longer detectable thereafter. This expression kinetics was similar to those of COL4A1 and LGR5 (Figure 5C). Furthermore, transfection of HepaRG progenitor cells with HAPLN1 siRNA downregulated the mesenchymal markers Snail, α-SMA, collagen type IV and the stem cell marker LGR5. Snail was also downregulated by β-catenin and LGR5-targeting siRNAs (Figure 6). Taken together, these findings indicate that HAPLN1 hallmarks mesenchymal commitment of hepatic progenitors.

### HAPLN1 is expressed at high levels in a subset of HCCs with bad outcome after curative resection

**Figure 7:**
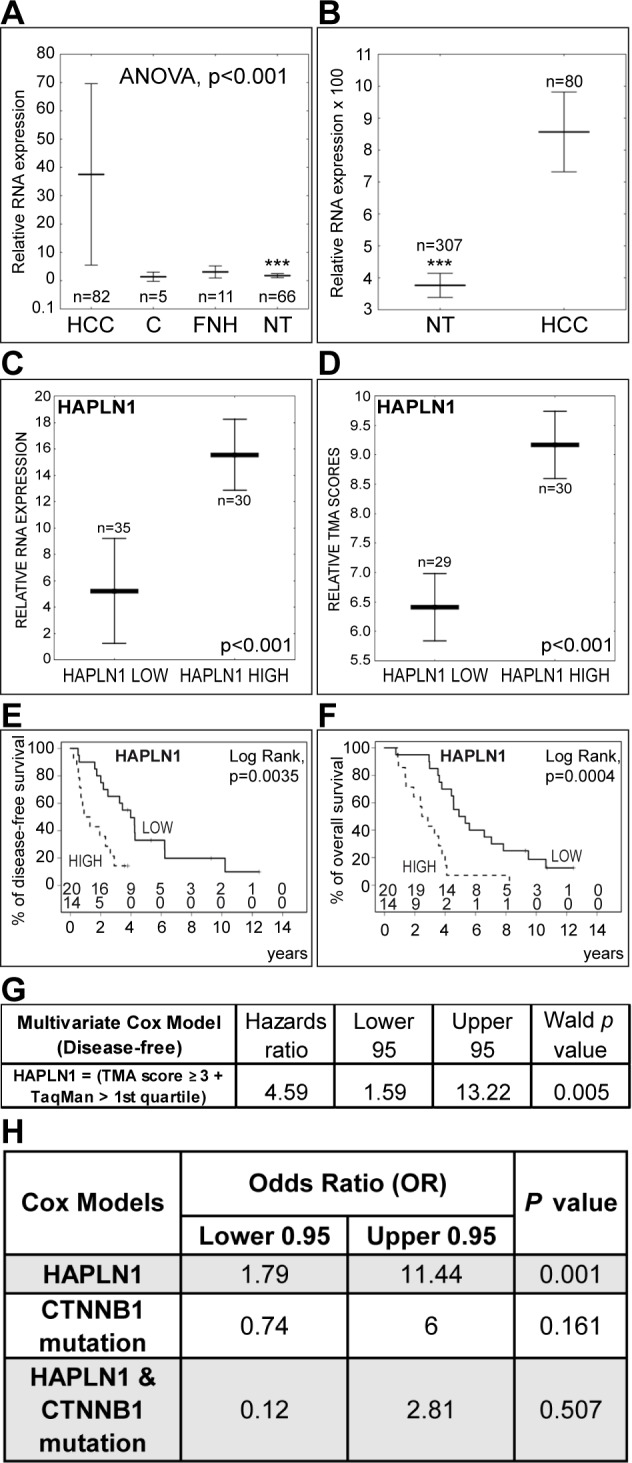
High HAPLN1 expression indicates bad outcome after curative HCC resection **A**. Real-time PCR analysis of HAPLN1 mRNA expression in HCC, histologically normal liver controls C, focal nodular hyperplasias FNH and matching non-tumor livers NT. Controls were used as calibrator. Asterisks (***) indicate *P*<0.001 for NT versus HCC. **B**. Comparison of HAPLN1 mRNA levels in HCC versus NT from a public DASL microarray dataset (GSE10143) [12] normalized by the cubic spline algorithm and filtered by fold change (up/down>3). **C**. and **D**. HAPLN1 LOW and HAPLN HIGH classes display different mRNA and protein levels by real-time PCR and by TMA-based immunohistochemistry scoring. **E**. and **F**. Disease-free and overall survival after curative HCC resection. The number of patients at risk at each time point is shown (*Upper row,* HAPLN LOW; *lower row,* HAPLN1 HIGH). **G**. Multivariate Cox's proportional hazards model after adjustment for alpha-fetoprotein, vascular invasion, number of resected liver segments and capsular infiltration. The model indicates that HAPLN1 is independently associated with survival. **H**. Cox's model integrating β-catenin (CTNNB1) exon 3 mutations shows that HAPLN1 is not dependent on β-catenin mutations.

HAPLN1 mRNA levels were measured by real-time PCR in 82 HCCs from 70 patients, 5 histologically normal liver controls, 11 FNHs and 66 matching non-tumor liver samples. HAPLN1 was detected at much higher levels in HCCs than in non-tumor livers in our cohort (Figure 7A), as well as in 80 HCCs with respect to 307 non-tumor hepatitis/cirrhosis livers in a public DASL microarray dataset (GSE10143) [12] (Figure 7B).

The clinical and biological features of the HCC patients in our cohort and the flowchart of inclusion criteria for survival analysis are detailed in Supplementary Table 8, A and B. A binary HAPLN1 expression score was generated from our real-time PCR and TMA immunohistochemistry data. *HAPLN HIGH* and *LOW* HCCs showed significantly different HAPLN1 mRNA levels and TMA immunohistochemistry scores (Figure 7C and 7D). *HAPLN HIGH* patients presented lower disease-free and overall survival (Figure 7E and 7F). Univariate Cox's analysis (Supplementary Table 9) confirmed the association of HAPLN1 with poor survival after curative resection. Multivariate analysis after adjustment for alpha-fetoprotein, vascular invasion, number of resected liver segments and capsular infiltration by the HCC showed that HAPLN1 was independently associated with survival after curative resection, with a mean increase in relative risk of 4.6 folds (Figure 7G). Of note, HAPLN1 relationship with survival was not dependent on β-catenin exon 3 mutations (Figure 7H) assessed by Sanger sequencing (Supplementary Table 10). Indeed, HAPLN1 expression was not driven by β-catenin activating mutations (Supplementary Figure 3). This contrasts with LGR5 (Supplementary Figure 3), which expression may be governed by both β-catenin mutations [1, 2] and by Wnt-driven stem cell amplification [13].

### HAPLN1 emerges in aggressive HCCs expressing markers of tumor stem/progenitor cells

**Figure 8:**
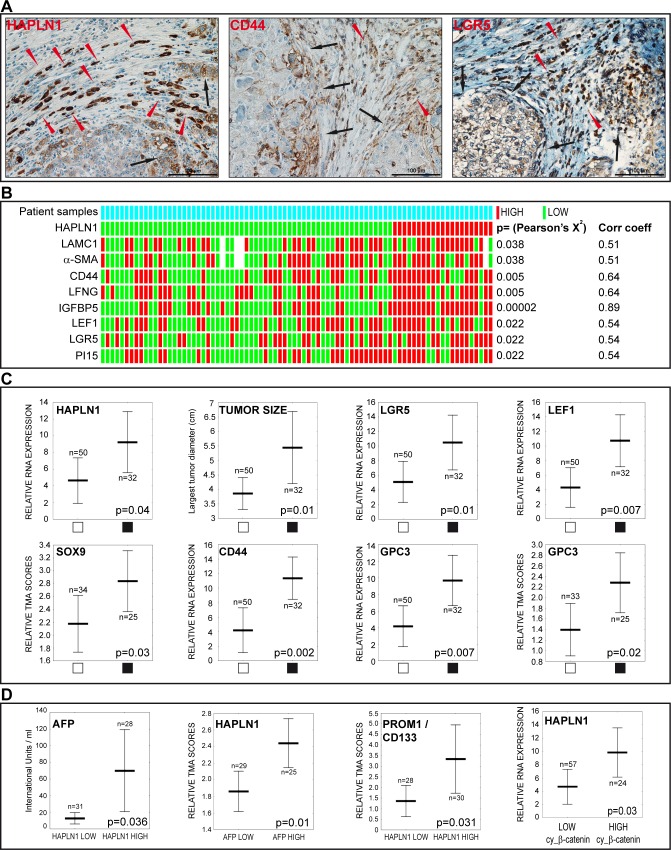
HAPLN1 is detected in HCCs featuring stem cell markers, high alpha-fetoprotein levels, vascular invasion and β-catenin activation **A**. Immunodetection of HAPLN1, CD44 and LGR5 in formalin-fixed, paraffin embedded HCCs. Brown color, specific signal; blue, hematoxylin counterstaining. The three proteins are detected in tumor cells at the tumor-stroma interface *(black arrows)* and in cells arranged in “Indian files” *(red arrowheads).* **B**. Heatmap showing associations of HAPLN1 mRNA expression with the indicated genes. White bars indicate missing data (n=8/729 spots). Pearson's X^2^ and Gamma correlation coefficients are shown on the right. **C**. Vascular tumor invasion *(black squares)* is associated with the indicated variables. **D**. Serum AFP levels are higher in *HAPLN1 HIGH* patients. High HAPLN1 protein correlates with high AFP serum levels. High PROM1/CD133 protein is detected in *HAPLN1 HIGH* HCCs. High HAPLN1 mRNA correlates with high cytoplasmic β-catenin.

HCCs showed co-expression of HAPLN1 with other genes found to be induced by Wnt3a in vitro. HAPLN1 and the tumor progenitor cell markers CD44 and LGR5 were detected in tumor cells at the tumor-stroma interface and in cells arranged in an “Indian file” pattern (Figure 8A). HAPLN1 correlated with laminin ɣ1, α-SMA, CD44, as well as with other genes induced by Wnt3a, such as LFNG, IGFBP5, LEF1 and PI15 (Figure 8B). HCCs with microscopic vascular invasion had high mRNA levels of HAPLN1, LGR5, LEF1, SOX9, CD44, Glypican 3 and larger tumor size (Figure 8C). Serum AFP levels were higher in *HAPLN1 HIGH* class patients and, similarly, patients with high AFP levels displayed high immunohistochemistry scores for HAPLN1. Likewise, high HAPLN1 expression correlated with the stem cell marker PROM1 and with cytoplasmic β-catenin (Figure 8D). Consistently, cytoplasmic β-catenin staining is increased in HCCs showing enhanced Wnt signaling without β-catenin gene mutations [1, 2]. Moreover, a cluster of biomarkers induced by Wnt3a in HepaRG cells reflecting progenitor cells with mesenchymal commitment was associated with high Edmondson-Steiner's score in HCCs, indicating poor tumor differentiation (Supplementary Figure 4). Taken together, these findings indicate that HAPLN1 emerges in mesenchymal-committed tumor progenitor cells driving tumor aggressiveness and poor outcome in HCC.

## DISCUSSION

**Figure 9:**
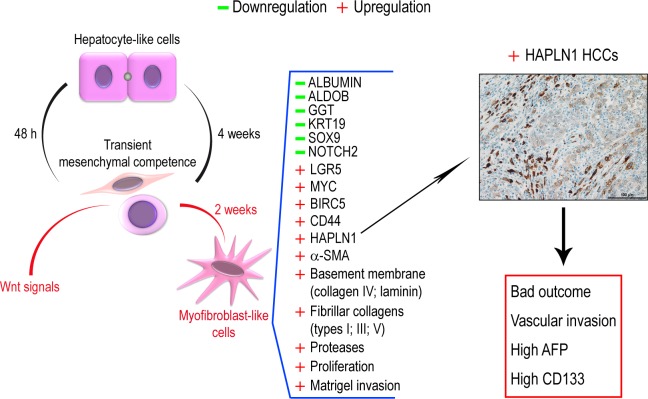
Upon low density plating, hepatocyte-like cells acquire transient mesenchymal competence within 48 h and dedifferentiate to bipotent progenitors that normally re-differentiate into hepatocyte-like cells A cell microenvironment enriched in Wnt signals reprograms liver progenitor cells into invasive fibrogenic myofibroblast-like cells within two weeks. These cells *de novo* express the cartilage gene HAPLN1 among other gene modulations. In HCCs, HAPLN1 is expressed *de novo* in a subset of patients presenting with HCCs that feature bad outcome, vascular invasion, high AFP serum levels and CD133+ tumor cells.

We show here that Wnt-specific signals foster fibrogenic, myofibroblast-like populations with stem cell features and impair hepatocyte and biliary lineage commitment of liver progenitors. The pathway crosstalks elicited by Wnt-specific signals form a tight network in HCCs. Among the genes switched on within this network, HAPLN1 is associated with bad outcome and with the expression of markers of stemness and of tumor aggressiveness (Figure 9).

We showed that Wnt3a upregulates TGFB1, TGFB2 and BMP1 expression. In turn, TGFB activates Wnt signaling and plays a key role in mesenchymal commitment [4, 27]. Thus, Wnt/TGFB pathways may set up a positive feedback loop leading to tumor aggressiveness. In addition, R-spondin binding of LGR5 receptors enhances Wnt signaling and leads to long-term expansion of bipotent hepatic progenitor cells [13]. In this context, excess of TGFB/BMP signaling is inhibited by Noggin, thus avoiding growth arrest and epithelial-mesenchymal transition [13, 28]. Our data suggest that Wnt3a triggers finely-tuned signaling pathway crosstalks, driving progenitor cell reprogramming to replicative, invasive and fibrogenic myofibroblast-like cells. Probably because Wnt3a upregulates Noggin (Figure 3A and 3F), as well as MYC and BIRC5 (Figure 1C), TGFB/BMP signaling is counterbalanced, thus avoiding growth arrest and achieving high proliferation rates throughout a two-week assay (Figure 1F). Despite their fibrogenic activity, these myofibroblast-like progenitors kept high levels of the stem cells markers CD44 and LGR5. Thus, the rates of proliferation and the expression of stem cell markers suggest that these cells are not terminally differentiated. This phenotype is compatible with the dynamics of cancer progression.

Wnt/β-catenin lies at the crossroads of a growing list of signaling pathways, including Hedgehog and Notch [5, 29]. High LGR5 expression was achieved through Wnt3a induction of Indian Hedgehog. As both Wnt [13] and Hedgehog [30] pathways upregulate LGR5 expression, Wnt/Hedgehog cross-talks probably amplified the LGR5^+^ stem cell population. Along these lines, Notch signaling leads to biliary lineage commitment [28], but Wnt3a reduced Notch2 expression (Figure 1C) and upregulated the Notch inhibitor LFNG (Figure 3A), thus impairing biliary lineage commitment. The Wnt-triggered signaling network also included insulin growth factor pathway members, namely IGFL1; 2 and 3, IGF2BP3 and FHL2. IGF2BP3 is an oncofetal IGF-mRNA-binding protein *de novo* expressed and promoting aggressiveness in HCC [31]. FHL2 enhances β-catenin-dependent transcription of IGFBP5 [32]. IGFBP5, in turn, increases the bioavailability of IGF1 and IGF2 [33] and induces fibrogenesis [34].

Activation of Wnt signaling was reported in hepatic stem cell-like HCCs expressing the epithelial cell adhesion molecule EPCAM [35]. We found that the cancer epithelial cell markers EPCAM and cytokeratin 19 (KRT19) were highly correlated in 79 HCCs (R=0.87, Supplementary Table 7). However, neither EPCAM nor KRT19 did integrate the coexpression network of mesenchymal-committed hepatic progenitors. Consistently, the earliest mesoderm-committed progenitor cells derived from human embryonic stem cells and mesenchymal-competent HepaRG progenitors are EPCAM− [18, 26]. As EPCAM^+^ HCC cells are epithelial [35], and our work is based on mesenchyme-competent EPCAM− hepatic progenitors [16, 18], we hypothesize that Wnt signals may direct transit-amplifying progenitor cells toward a mesenchymal phenotype. Importantly, through activation of different gene networks, both works show that the final output is enhanced tumor aggressiveness.

Wnt signals led cells to ectopically express mesenchymal progenitor genes like HAPLN1, which regulates cell growth in developing cartilage [19] and heart valves [36]. Similarly, IGFBP5 and PI15 are involved in branching morphogenesis of epithelial organs [37]. In addition, HAPLN1 was expressed *de novo* upon mesenchymal commitment of embryonic stem cells and in two models of liver EMT. Conversely, HAPLN1 knockdown in hepatic progenitor cells downregulated LGR5, α-SMA, collagen IV and Snail. In HCCs, in consistency with *in vitro* data, high HAPLN1 levels were associated with basement membrane remodeling, myofibroblast activation and expression of the stem/progenitor markers CD44, LGR5 and CD133/PROM1; the Notch pathway inhibitor LFNG, the Wnt target gene LEF1 and the IGF pathway agonist IGFBP5 (Figure 8). Supporting our data, a bioinformatics study predicted HAPLN1 expression to lie downstream upregulated Wnt/β-catenin signaling in colorectal cancer [38] and proteomics analysis of tumor stroma reported HAPLN1 in highly metastatic melanomas [25]. Also, HAPLN1 has been associated with bad outcome in pleural mesothelioma [39].

In conclusion, Wnt-specific signals led hepatic progenitors to specialize into invasive, proliferating myofibroblast-like fibrogenic cells with stem cell features through a subtle equilibrium of signaling pathway crosstalks. Through the ectopic induction of mesenchymal progenitor genes in liver cancer cells, Wnt signals confer a high degree of biodiversity and, ultimately, selective advantages for cancer progression.

## MATERIALS AND METHODS

### Patients, clinical data management and tissue banking

Seventy HCC patients undergoing liver surgery with curative intention at Rennes University Hospital between January 1992 and December 2007 were included. Fifty-one were treated by partial hepatectomy and 19 by orthotopic liver transplantation (OLT). The microscopic features of tumors diagnosed as HCC were reviewed and annotated by a senior pathologist (BT). Combined hepatocellular-cholangiocarcinoma or fibrolamellar HCCs were not included. Demographic, clinical and biological data were retrieved from hospital charts by two experienced liver surgeons (LS & DB) and are described in Supplementary Table 8A. After resection, patients were followed up every 3 months for the first 2 years, and every 6 months thereafter. Follow-up, which was available for 65 patients, included clinical examination, serum AFP (alpha-fetoprotein), ultrasonography and computed tomography. Mean and median follow-up times were 60 and 53 months, respectively (95% CI= 50-70 months; range= 3-150 months). The end of follow-up was set between June and September 2012 or at the time of death. Only patients undergoing partial hepatectomy, with a follow-up ≥ 3 months and with complete tumor resection were included in the survival analyses. Early deaths, unrelated to the HCC, and patients without microscopically tumor-free resection margins, as well as patients treated by OLT, were excluded from survival analyses. Survival analysis flowchart is shown in Supplementary Table 8B. Eighty-two frozen tumors from 70 patients were analyzed. As controls, we included 66 matching non-tumor livers, 5 histologically normal livers from patients undergoing resection of liver metastases of extrahepatic primary cancers and 11 focal nodular hyperplasias. Non-tumor and normal liver samples were obtained at >2 cm from the tumors. Frozen human tissue samples were obtained after processing by the Biological Resource Center (BRC) at Rennes University Hospital (BB-0033-00056). Briefly, fresh tissues were frozen at −80°C in N_2_-cooled isopentane and stored at −80°C under quality-controlled conditions. Formalin-fixed, paraffin-embedded tissue blocks were obtained from the Anatomic Pathology laboratory. The study protocol complied with French laws and regulations and fulfilled the requirements of the local institutional ethics committee. Sample collection was reported to the Ministry of Education and Research (No. DC-2008-338).

### Cell culture

Huh-7 [8] and 3P/3SP [23] cells were cultured [10] and Wnt3a-enriched conditioned medium was collected as described [9, 10]. HepaRG cells were expanded as liver progenitors, differentiated to hepatocytes and retrodifferentiated to liver progenitors [18]. To induce EMT in HepaRG cells, progenitor HepaRG cells were detached by trypsinization, seeded at low density (2×10^4^ cells/cm^2^), detached three days later and seeded at the same density in complete HepaRG medium containing 50% Wnt3a-conditioned or 50% control L cell medium. In other experiments, purified mouse recombinant Wnt3a (R&D Systems, 7nM) and/or mouse FZD8_CRD (30 nM, R&D Systems) were added to serum-free HepaRG medium containing 0.1% BSA for the indicated periods of time according to the experiment. Independent culture experiments were performed in triplicate.

### Tissue-microarray (TMA) and immunohistochemistry scoring of human tissues

For selection of tissue blocs for TMA construction, the whole set of archival HE-stained sections from formalin-fixed, paraffin-embedded tissue blocks from each one of the 67 available HCC patients and from five histologically normal livers were reviewed (Nikon 80i microscope) to ensure that the selected paraffin bloc was the mirror lesion of the frozen sample. A region of interest (ROI, 0.5 to 1 cm in diameter) was labeled with a permanent marker to precisely localize the punch zone. TMA construction was done with a MiniCore3 tissue arrayer (Alphelys, Plaisir, France). ROIs were punched in triplicate. Punch size was set at 1000 μm in diameter. HCC samples were randomly assigned to either one of two 132-punch receiver paraffin blocks. Randomization was performed with a *“rand”* function in Microsoft Excel. The five histologically normal liver controls were included in both array blocs #1 and #2 to control for signal and background intensities. Five-μm microtome sections were processed for immunohistochemistry with a Discovery XT from Ventana Medical Systems (Roche) slide staining system. See Supporting Table 2 antibodies used. Stained slides were converted into high-resolution digital data with a NanoZoomer digital slide scanner (Hamamatsu, Massy, France). Digital slides were viewed using NDP.view software (Hamamatsu) in a Dell S2415H 24-Inch Screen LED-Lit Monitor. The signal was independently read by two observers (RD and OM) who were blinded to any information. Discrepancies were resolved by consensus reading. A 4-point scale (0-1-2-3-4) denoting increasing signal intensity was used. The relative importance of focal versus general expression of the proteins under study was weighted using the following formulas, where *GS* stands for general score and *FS* stands for focal score:

If general signal predominated, *score = 2xGS + FS + 1*; (where +1 was used to weight the relative importance of general signal with respect to focal signal).

If focal signal predominated, *score = 2xFS + GS*.

When applied, these formulas yielded scores varying from 3 to 13.

### Cell growth assays

Cells were seeded at low density (5×10^3^ cells per well in 96-well plates). HepaRG culture medium was supplemented with 5-bromo-2′-deoxyuridine (BrdU, GE Healthcare Lifesciences) for 1h30 and cells were fixed with 4% PFA. BrdU was detected with mouse anti-BrdU antibody (1:200, ab8152; Abcam) followed by anti-mouse IgG-DyLight 488 (1:500, 072-03-18-18, Eurobio, Les Ulis, France). Image acquisition and quantitation of BrdU-incorporating cells were performed with a Cellomics ArrayScan VTI HCS Reader (Thermo Scientific).

### Cell invasion

Cell invasion was assessed in Matrigel-coated Boyden chambers (Transwell inserts, 8.0 μm pore size, Corning). After five days of culture following a routine passage, cells were detached, washed with PBS and suspended in serum-free medium supplemented with 0.1 % BSA. Then, 1×10^5^ cells were seeded in the upper chamber of the Transwell insert. The lower chamber was filled with 800 μl of serum-free medium with or without recombinant mouse Wnt3a (R&D Systems). After 24 hr of incubation at 37°C, cells were fixed with 4% PFA and stained with 2.3% crystal violet. Non-invading cells were gently removed from the upper surface of the insert and images from five fields per membrane were randomly acquired (Axio observer, Carl Zeiss microscopy). Quantitation of invading cells was done with ImageJ (NIH).

### β-catenin exon 3 sequencing and search for β-catenin exon 3 deletions

For mutation analysis, exon 3 and the flanking intronic sequences of the CTNNB1 gene were amplified by PCR using Kappa HiFi DNA polymerase (Kapa Biosystems, Wilmington, MA). Briefly, 50 ng of genomic DNA were used as template with βCATEX2F (5′-GAA-AAT-CCA-GCG-TGG-ACA-ATG-3′) and βCATEX4R (5′-TCG-AGT-CAT-TGC-ATA-CTG-TCC-3′) as oligonucleotide primers. The reaction included a 95°C denaturation step for 3 min followed by 35 cycles consisting of: 98°C denaturation for 15 sec, 60°C annealing for 15 sec, and elongation 72°C for 30 sec. Both strands of the amplified products were directly sequenced on an automated sequencer (ABI 3700; Perkin-Elmer, Boston, MA). Negative results on exon 3 mutations may overlook large exon 3 deletions in CTNNB1. Indeed, as somatic deletions are heterozygous, the wild-type allele may mask the deletion upon Sanger sequencing [40]. To avoid this pitfall, cases showing no exon 3 mutation were screened for *β-catenin* deletion by PCR on reverse-transcribed tumor cDNA. For PCR amplification of the exon 3 of CTNNB1, oligonucleotide primers (F1: 5′- GCG-TGG-ACA-ATG-GCT-ACT-CAA-G-3′; R1: 5′- TAT-TAA-CCA-CCA-CCT-GGT-CCT-C-3′; and R2: 5′- TTC-AGC-ACT-CTG-CTT-GTG-GTC-3′) were used. The primer pairs F1-R1 and F1-R2 yielded 517 bp (169 aa) and 1043 bp (344 aa) amplicons, respectively. As positive control, HepG2-derived cDNA was used, as these cells harbor a large CTNNB1 deletion (amino-acids 25-140) [40]. PCR was performed using the GoTaq Green Master Mix (Promega) and amplicons migrated in 1.5% Nusieve agarose (LifeTechnologies) gels.

### DNA microarrays

In vitro transcription and labeling of 150 ng total RNA was carried out using an Agilent Low-Input QuickAmp Labeling kit (Agilent Technologies, Santa Clara, CA, USA) in the presence of Cy3-CTP following the manufacturer's protocol. After purification using an RNeasy mini kit (Qiagen, Hilden, Germany), cRNA yield and specific activity were determined using a NanoDrop spectrophotometer, as previously described [10]. Equal amount of Cy3-labeled cRNA was subjected to fragmentation followed by 18-hr hybridization onto Agilent human 4×44K v2 pangenomic microarrays. Washing and scanning were performed according to the manufacturer's instructions (Agilent Technologies). Gene expression data were further processed using Feature Extraction (version 10.7) and GeneSpring (version 11.0) softwares (Agilent Technologies). Filtration of array data resulted in the selection of nonflag-positive and significant gene features. Inter-array normalization was performed by using the 75th percentile signal value. Transcriptomic data have been deposited in NCBI's Gene Expression Omnibus and are accessible through GEO Series accession number GSE68633.

### Functional genomics analyses

Hierarchical clustering was performed with the *hclust* function in R software, applying the Ward method to correlation-based distances. Gene annotations based on gene ontology and enrichment for biological functions were done with the FuncAssociate 2.1 program [21]. Gene ontology functions were summarized by removing redundant terms using REVIGO [41]. Ingenuity pathway analysis was used to identify top biological functions, upstream regulators and transcription regulators (Ingenuity Systems, Redwood City, CA, USA). Gene set enrichment analysis (GSEA) was performed with the web-based tool developed at the Broad Institute [42].

### Statistical analyses

After removing experimental batch effect with the *SVA* package [43], gene networks were generated by Weighted Correlation Network Analysis (WGCNA, WGCNA package) [44] using expression data. Gene networks were visually integrated with Cytoscape, with a correlation coefficient threshold ≥0.30 [45]. To analyze patient data, the Recommendations for Tumor Marker Prognostic Studies (REMARK) were applied [46]. Briefly, to screen our transcriptomic dataset for biologically relevant genes warranting further studies, we focused on those meeting two criteria: (i) response to both β-catenin targeting siRNA and FZD8_CRD in independent experiments and (ii) involvement in key signaling pathways. Eight genes meeting these criteria were analyzed by real-time PCR and TMA-based immunohistochemistry in human HCCs. Complying with the REMARK guidelines [46], the inclusion criteria for survival analysis are shown by a flowchart in Supplementary Table 8B. Survival analyses were performed using the Log Rank test and Kaplan-Meier curves and the Cox's proportional hazards method for univariate or multivariate analyses, as indicated. To this end, real-time PCR results were calculated by the ΔΔCt method and calibrated with respect to the median of a standard range curve run in every experiment. Real-time PCR results were further normalized by the following equation: *[(LNi-mean_LNie→n_)/SD_LNi→n_]*, where *LN,* natural logarithm; *i→n,* from the first to the last value. To obtain binary values for Kaplan-Meier curves, normalized real-time PCR results were binarized using the median as the cut-off value. TMA-based immunohistochemistry scores (0-1-2-3-4) were binarized by a ≥3 cut-off score for focal expression of specific proteins.

Correlation analyses between gene expression levels were performed with the Spearman rank order test (continuous variables, non-parametric distribution). Association analyses between binary or ordinal variables (expression scores, clinical, biological or anatomic pathology data) were performed with Pearson's Chi2 or Fisher exact test. Group comparisons were done with non-parametric ANOVA and the Kruskal Wallis post hoc tests, unless otherwise indicated. Heatmaps showing associations between binary patient data were constructed with Excel (Microsoft Office 2010). Statistical calculations were performed with the R software (version 3.1.1) and the packages *survival, FactoMineR, gplots, colorspace* and *marray* or with Statistica 10 (Statsoft, Maisons-Alfort, France).

See Supplementary Materials and Methods for: RNA interference, nucleic acid extraction from human tissues and cells, real-time PCR and immunocytochemistry. See Supplementary Table 1 for real-time PCR primers and TaqMan probes and Supplementary Table 2 for antibodies.

## SUPPLEMENTARY MATERIALS FIURES AND TABLES







## Abbreviations

α-SMA: alpha smooth muscle actin
AFP: alpha-fetoprotein
APC: adenomatous polyposis coli
BRC: biological resource center
CTNNB1: DASLc, DNA-mediated Annealing, Selection extension, and Ligation Assay
EMT: epithelial-mesenchymal transition
FNH: focal nodular hyperplasia
FZD: frizzled
GGT1: gamma-glutamyltransferase 1
GSE: gene expression series
GSK3B: glycogen synthase kinase 3 beta
HAPLN1: hyaluronan and proteoglycan link protein 1
HCC: hepatocellular carcinoma
IGFBP5: insulin growth factor binding protein 5
LEF1: lymphoid enhancer-binding factor 1
LFNG: lunatic fringe homolog
MMP2: matrix metallopeptidase 2
NT: non-tumor
OLT: orthotopic liver transplantation
PI15: peptidase inhibitor 15
PROM1: prominin 1 (CD133)
SFRPs: secreted frizzled-related proteins
siRNA: small interfering RNA
T: Tumor
TGFB: transforming growth factor beta.
