# Supplementary material for: De novo HAPLN1 expression hallmarks Wnt-induced stem cell and fibrogenic networks leading to aggressive human hepatocellular carcinomas
Source: Oncotarget. 2016 May 13;7(26):39026–43. doi: 10.18632/oncotarget.9346 (PMC5129911; doi:10.18632/oncotarget.9346)
Supplement: Supplementary file 1 [file oncotarget-07-39026-s001.pdf]

De novo HAPLN1 expression hallmarks Wnt-induced stem cell and fibrogenic networks leading to aggressive human hepatocellular carcinomas

Supplementary Material

This file includes:

Page number: 24,

Table of contents,

Supplementary Materials and Methods,

Supplementary Figures (with legends below each figure): 4,

Supplementary Tables: 10,

References: 6.

Table of contents

Supplementary materials and methods

RNA interference and cDNA vector transfection 4

Nucleic acid extraction from human tissues and from in vitro cultures 4

Real-time PCR 5

Immunocytochemistry 5

Supplementary figures

Supplementary Figure 1 6

Supplementary Figure 2 8

Supplementary Figure 3 10

Supplementary Figure 4 11

Supplementary Tables

Supplementary Table 1 13

Supplementary Table 2 14

Supplementary Table 3 (Transcriptomic data, to be found as a separate excel file)

Supplementary Table 4 (Transcriptomic data, to be found as a separate excel file)

Supplementary Table 5 (Transcriptomic data, to be found as a separate excel file)

Supplementary Table 6 15

Supplementary Table 7 21

Supplementary Table 8 (Transcriptomic data, to be found as a separate excel file)

Supplementary Table 9 (Transcriptomic data, to be found as a separate excel file)

Supplementary Table 10 23

References to Supplementary data 24

Supplementary Materials and Methods

RNA interference and cDNA vector transfection

Routinely expanded HepaRG progenitor cells were detached by trypsinization, seeded at low density (2x10^4 cells/cm^2), detached three days later and transfected in suspension with small interfering RNAs (siRNAs) by a MP100 Microporator (LabTech) and Neon™ Transfection System 100 µL Kit (Invitrogen) under optimized conditions (80 pmoles siRNA, 1500V, 20ms, 1 pulse). Silencer negative control (D-001810-10-05, Thermo Fisher) and β-catenin (HSS102460, Invitrogen) siRNAs were used.

Nucleic acid extraction from human tissues and from in vitro cultures

RNA and DNA were extracted from 10 µm cryosections obtained from frozen tissue blocks. Once tissues were placed on the specimen head of a Leica 3050S cryostat and trimmed to obtain a level and histologically controlled representative surface (hematoxylin or toluidine blue extemporaneous staining), the first two 5 µm sections were set aside as permanent histological controls. Then, 10 µm cryosections were obtained and immediately lysed in RNA or DNA extraction buffers. Before removing the disk containing the specimen from the head, two 5 µm cryosections were set aside again as permanent histological controls. Lead and rear permanent histological controls were formalin-fixed, HE-stained in a Leica ST5020 routine stainer and representativity was assessed by two independent observers (BT & OM). Non-representative samples were removed from the RNA and DNA extraction process. Representative samples were extracted with NucleoSpin® RNA II (Macherey-Nagel) and with the DNeasy Blood & Tissue (Qiagen) kits, as recommended by the manufacturers. Quality control of the extracted nucleic acids

was performed by dosing the eluents with a NanoDrop spectrophotometer and by checking 28S/18S ratios by gel electrophoresis. For in vitro cultured cell layers, total RNA was purified with an RNAeasy kit (Qiagen). Extracted RNA quality control was done as for human tissues.

Real-time PCR

Reverse-transcribed cDNA was obtained with the High Capacity cDNA Reverse Transcription Kit (Applied Biosystems) according to manufacturer's instructions. Expression of relevant genes was measured by quantitative real-time PCR using the StepOnePlus™ System or the ABI prism 7900HT, power SYBR Green PCR master mix or Taqman probe-based assays (Applied Biosystems), according to the experiments. See Supporting Table 1 for real-time PCR primers and TaqMan probes. Quantitative analyses of PCR data were conducted by the $2^{-\Delta\Delta Ct}$ method.

Immunocytochemistry

Cells were fixed with 4% paraformaldehyde (PFA) for 15 minutes at room temperature (RT) and permeated in 0.1 % Triton-X-100 (Sigma) for 15 min at RT. Primary antibodies used are listed in Supporting Table 2. Nuclei were stained with DAPI. Secondary antibodies were: anti-mouse FITC-labeled (F8521, Sigma Aldrich), anti-rabbit Cy5-labeled (A10523, Life Technologies) and anti-goat FluoProbes® 594 (FP-SD2110, Interchim). Images were acquired using a Cellomics ArrayScan VTI HCS Reader (Thermo Scientific).

Supplementary Figures:

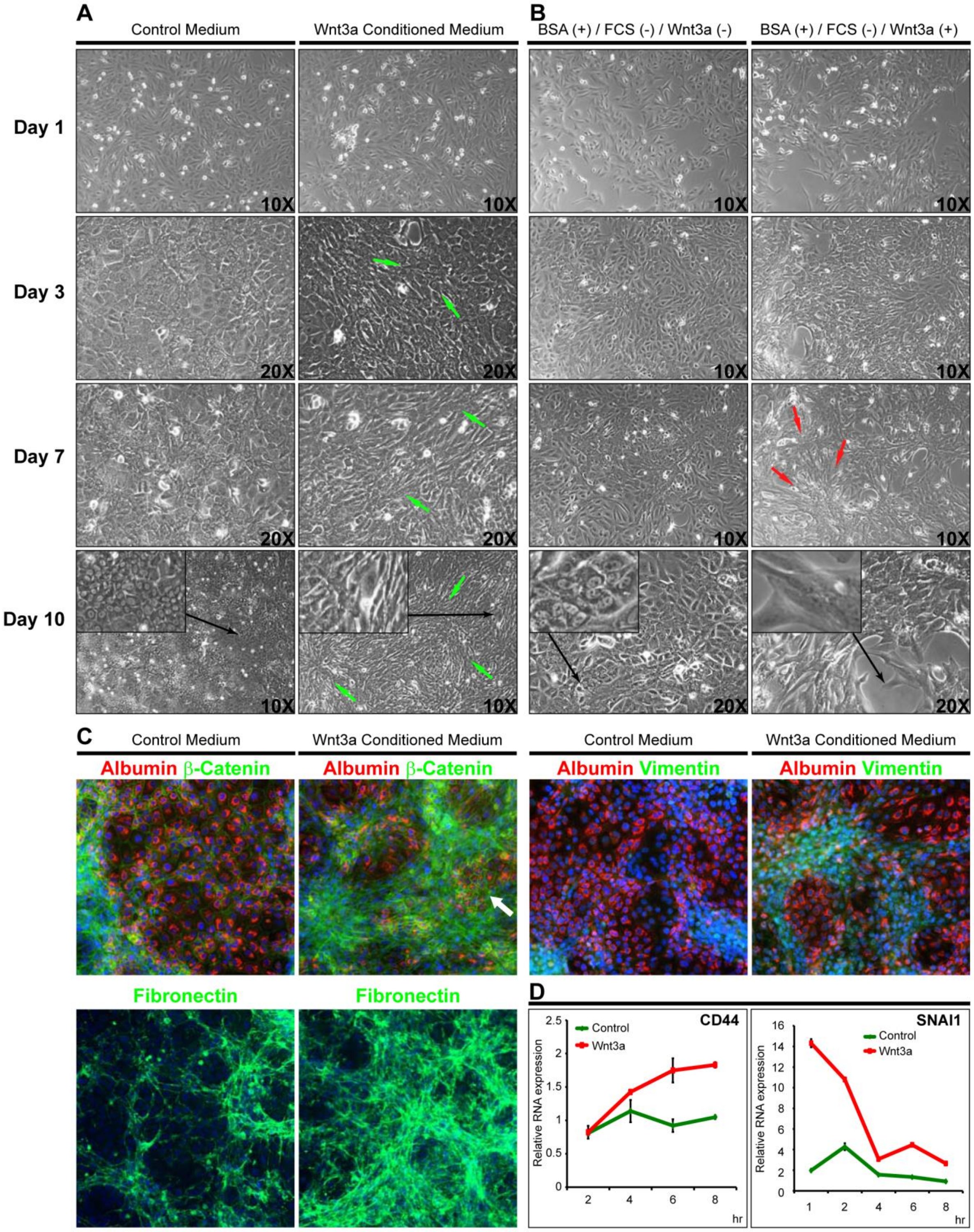

Supplementary Figure 1: Wnt3a induces fibrogenic myofibroblast-like cells from liver cell progenitors. A. Ten-day kinetics of incubation of progenitor HepaRG cells with control versus Wnt3a conditioned medium containing 10% FCS or with **B.**

FCS-free control medium versus FCS-free medium plus 7 nM purified recombinant Wnt3a (R&D Systems). Both purified recombinant Wnt3a and in-house produced conditioned medium from Wnt3a-secreting cells (CRL-2647, ATCC) induce similar morphological changes in progenitor HepaRG cells over 10 days. Cells receiving control media gradually develop the features of a hepatocyte culture (see insets for cytological details). Wnt3a treatment leads to the progressive growth of fascicles of spindle cells with elongated nuclei. Transition from a hepatocyte-like to a fibroblast-like morphology is particularly evident at day 3, when polygonal, elongated hepatocyte-like cells with granular cytoplasm and round nuclei coexist with fusiform cells arranged in parallel fascicles (green arrows). At day 7, closely packed spindle cells are arranged in parallel (green arrows) or intersecting (red arrows) fascicules. At day 10, densely packed whorls of spindled fibroblast-like cells show a storiform-like pattern (green arrows). At high power (insets) these cells show clear elongated nuclei, parallel to the cell axis. **C.** Coimmunodetection of the indicated proteins in HepaRG cells after 13 days of incubation with Wnt3a-conditioned or control media. β-catenin, Vimentin and fibronectin: green (FITC), Albumin: red (FluoProbes 594 and Cy5, respectively), nuclei: blue (DAPI). Control cells show large foci of Albumin (+) hepatocytes with membranous β-catenin. Wnt3a-incubated cells are arranged in bridging fascicules of spindle β-catenin (+)/Vimentin(+)/fibronectin(+)/Albumin(-) cells surrounding and overlaying membranous β-catenin(+)/Albumin(+) (arrow) foci. Color-merged images were acquired with a Cellomics cellular imaging station at 20X. **D.** Kinetics of mRNA expression of CD44 and SNAI1 analyzed by real-time PCR in progenitor HepaRG cells upon Wnt3a-induced EMT. Cells were cultured with control FCS(-)/BSA(+) or FCS(-)/BSA(+)/Wnt3a(+) media as indicated.

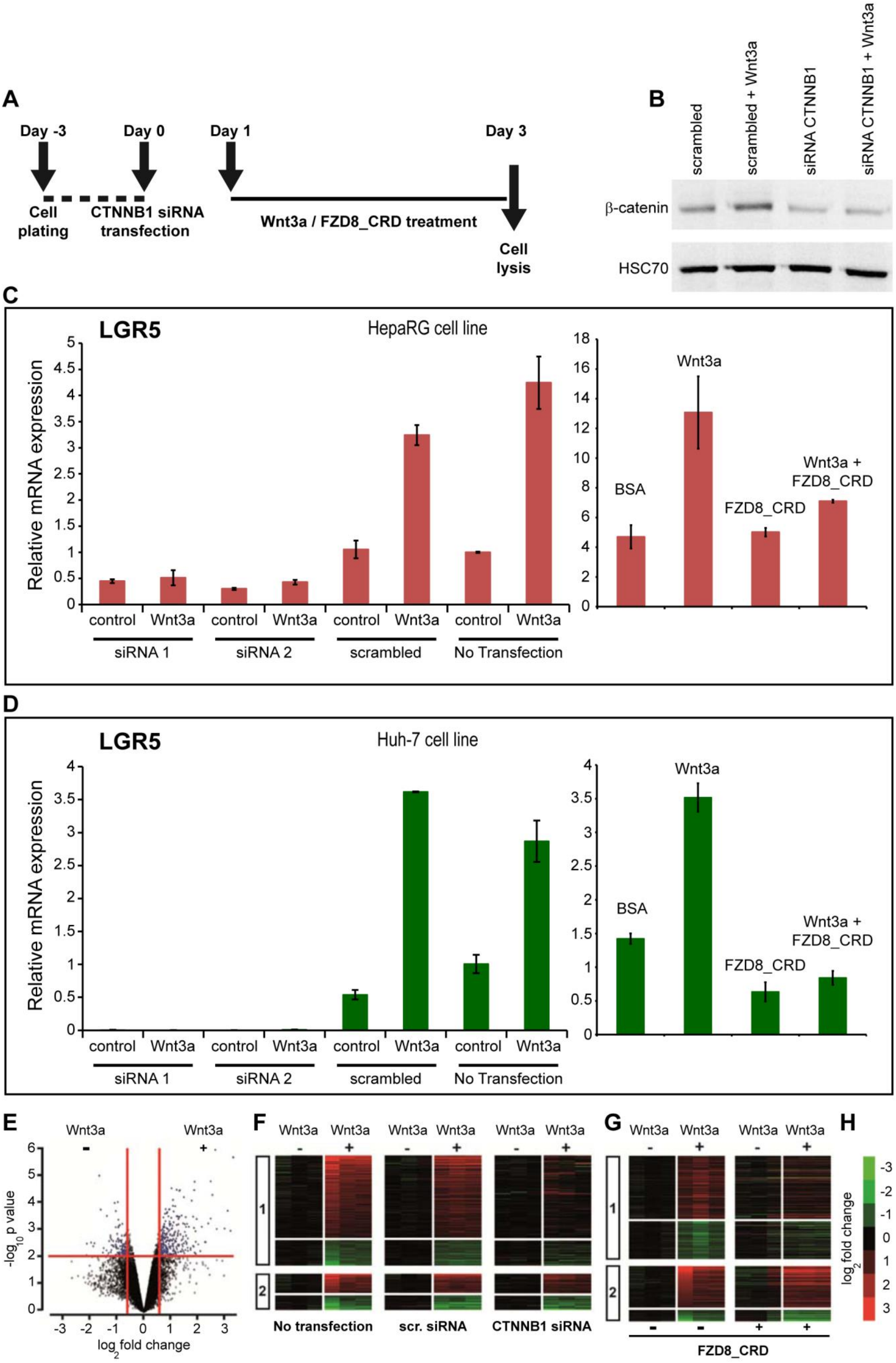
A
Day -3
Day 0
Day 1
Day 3
Cell plating
CTNNB1 siRNA transfection
Wnt3a / FZD8_CRD treatment
Cell lysis
B
scrambled
scrambled + Wnt3a
siRNA CTNNB1
siRNA CTNNB1 + Wnt3a
β-catenin
HSC70
C
LGR5
HepaRG cell line
Relative mRNA expression
control
Wnt3a
siRNA 1
siRNA 2
scrambled
No Transfection
BSA
Wnt3a
FZD8_CRD
Wnt3a + FZD8_CRD
D
LGR5
Huh-7 cell line
Relative mRNA expression
control
Wnt3a
siRNA 1
siRNA 2
scrambled
No Transfection
BSA
Wnt3a
FZD8_CRD
Wnt3a + FZD8_CRD
E
Wnt3a -
Wnt3a +
-log10 p value
log2 fold change
F
Wnt3a -
Wnt3a +
No transfection
scr. siRNA
CTNNB1 siRNA
G
Wnt3a -
Wnt3a +
FZD8_CRD
H
log2 fold change

Supplementary Figure 2: Modulation of the transcriptome profile of Wnt3a-induced fibrogenic myofibroblast-like cells by β-catenin targeting siRNA and by the Wnt inhibitor FZD8_CRD.

A. Experimental design. HepaRG cells were plated three days before transfection of β-catenin-targeting siRNA and treated with 7 nM Wnt3a alone and/or 30 nM FZD8_CRD from day one through day three after transfection, as indicated. **B.** Immunoblot showing β-catenin stabilization with soluble Wnt3a and silencing with β-catenin targeting siRNA. HSC70 is used as a loading standard. **C** and **D.** Application of the experimental protocol described in *A* to real-time PCR analysis of LGR5 mRNA expression in HepaRG ***C*** and Huh-7 ***D*** cells treated with Wnt3a after transfection with β-catenin targeting or control (scrambled) siRNA or no transfection *(left)* or after treatment with Wnt3a alone and/or FZD8_CRD *(right)*. **E.** Volcano plot of 358 genes differentially expressed in HepaRG cells cultured with (+) or without (-) recombinant Wnt3a for 72 h (n=3 independent experiments). Gene filtering is based on the significance of the differential gene expression following Wnt3a treatment (horizontal red line; $p<0.01$) and fold change (vertical red lines; >1.5 or <0.7). **F.** Expression profiles of genes differentially regulated by Wnt3a in cells that were not transfected (No transfection) (left panel), transfected with a control scrambled siRNA *(scr. siRNA,* middle panel) or with a β-catenin-targeting siRNA (right panel). To obtain β-catenin-dependent genes, 169/358 Wnt3a-modulated genes affected by control scrambled siRNA transfection were filtered out. Two gene clusters are shown: *1* (upper panel), 142 genes whose responses to Wnt3a were inhibited by β-catenin siRNA (β-catenin-dependent Wnt3a signature) and *2* (lower panel), 47 genes whose responses to Wnt3a were not affected by β-catenin siRNA (β-catenin-independent Wnt3a signature). **G.** Expression profiles of 358 genes differentially modulated by Wnt3a

alone and/or FZD8_CRD, as indicated. Two gene clusters are shown: *1* (upper panel), 233 whose responses to Wnt3a were inhibited by FZ8_CRD (FZD8_CRD-sensitive signature) and *2* (lower panel), 125 genes whose responses to Wnt3a were not affected by FZ8_CRD (FZD8_CRD-refractory signature). **H.** Heatmap color key.

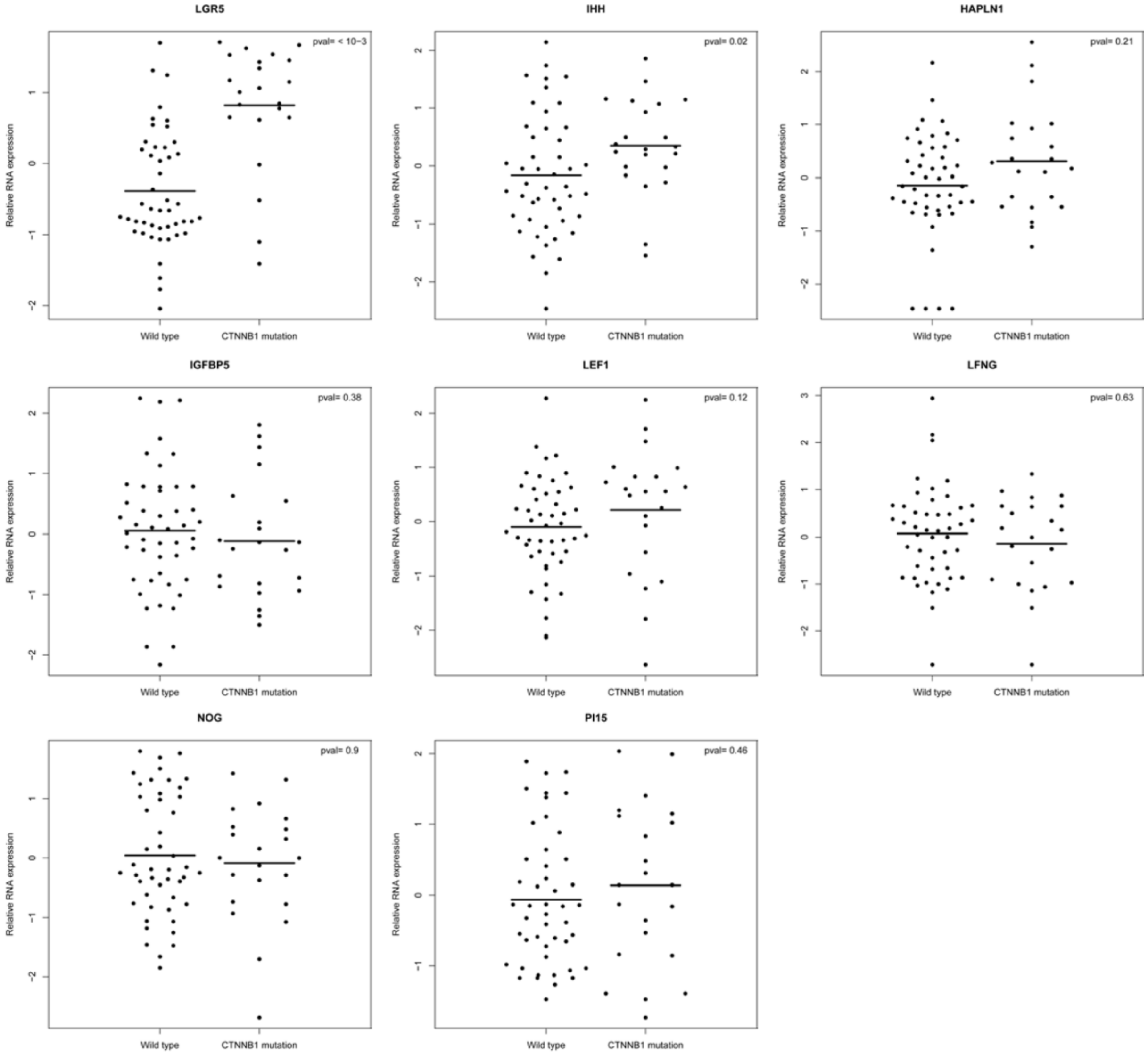

Supplementary Figure 3: Real-time PCR expression of the indicated genes' mRNA levels in HCCs carrying wild-type or mutant β-catenin as assessed by Sanger sequencing of CTNNB1 exon 3.

A

Genes correlated with IGFBP5			
GENE ID	n=	R=	*P*
CD44	82	0.42	<0.001
SOX9	82	0.50	<0.001
ACTA2	78	0.25	0.02
LGR5	82	0.25	0.02
PI15	82	0.40	<0.001
LFNG	82	0.70	<0.001
LEF1	82	0.60	<0.001
COL4A1	78	0.34	0.002

Genes correlated with LFNG			
GENE ID	n=	R=	*P*
CD44	82	0.47	<0.001
SOX9	82	0.47	<0.001
COL4A1	78	0.37	<0.001
LEF1	82	0.57	<0.001

Genes correlated with PI15			
GENE ID	n=	R=	*P*
CD44	82	0.47	<0.001
LGR5	82	0.48	<0.001
LEF1	82	0.50	<0.001

Genes correlated with LEF1			
GENE ID	n=	R=	*P*
LGR5	82	0.55	<0.001
CD44	82	0.53	<0.001
GPC3	82	0.40	<0.001

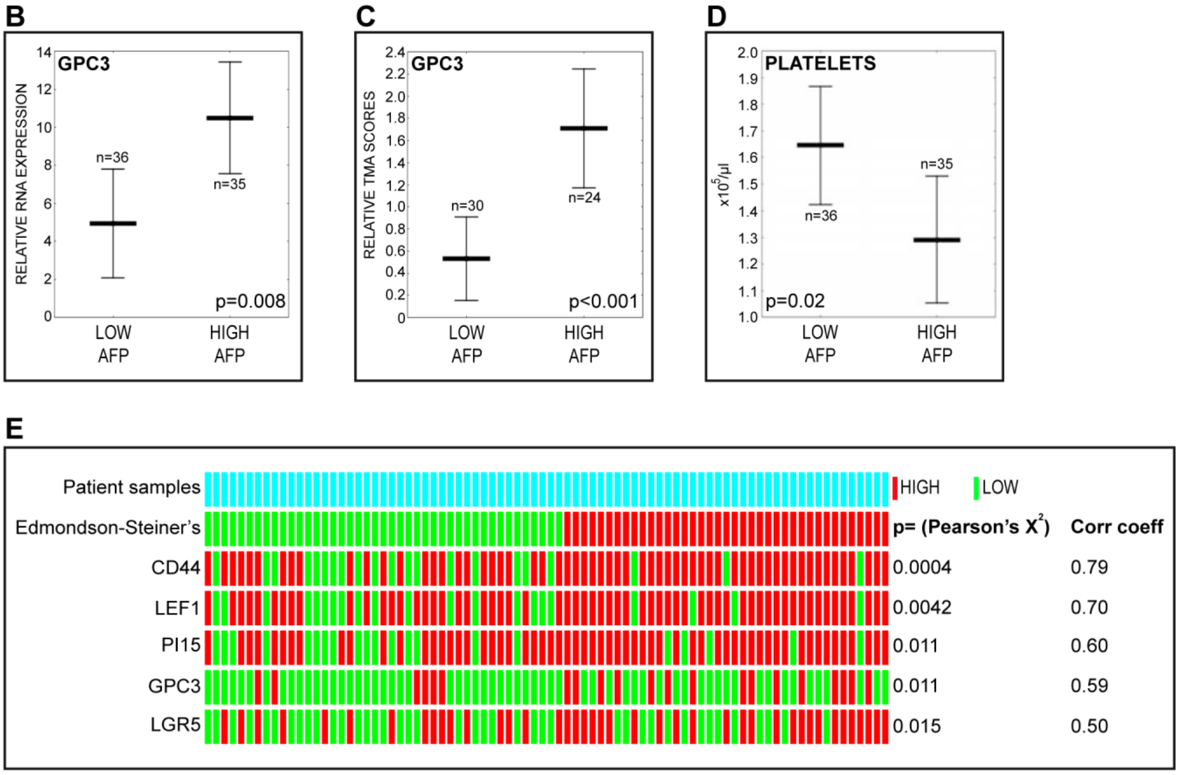

Supplementary Figure 4: Biomarkers induced by Wnt signals in vitro are associated with low tumor differentiation in HCCs.

A. Gene expression was assessed by real-time PCR. *R=,* Spearman's rank order correlation coefficients; *n=,* number of HCCs. **B.** High serum alpha-fetoprotein levels (cut-off > median value of 6.3 IU/ml) are associated with GPC3 mRNA levels analyzed by real-time PCR, **C.** with GPC3 protein levels analyzed by TMA

immunohistochemistry scoring and **D.** with low platelet counts. **E.** The heatmap plot shows that poor cytoarchitectural differentiation of HCCs (high Edmondson-Steiner's scoring, cut-off, ≥3) is associated with the mRNA expression levels of the indicated genes. Pearson's X^2 and Gamma correlation coefficients are shown on the right.

Supplementary Table 1: (A) Sybr Green primers and (B) TaqMan probes.

Supplementary Table 1A

SYBR GREEN PRIMERS (Eurogentec, Seraing, Belgium)		
**GENE ID**	**STRAND**	**Primer sequences 5'- 3'**
ACTA2	Forward	CATCCTCATCCTCCCTTGAG
	Reverse	ATGAAGGATGGCTGGAACAG
ALDOB	Forward	GCATCTGTCAGCAGAATGGA
	Reverse	TAGACAGCAGCCAGGACCTT
BIRC5	Forward	TCCGGTTGCGCTTTCCT
	Reverse	TCTTCTTATTGTTGGTTTCCTTTGC
PROM1	Forward	ACACTACCAAGGACAAGGCG
	Reverse	TGATGTTGGGTCTCAGTCGG
CD44	Forward	GGCTTTCAATAGCACCTTGC
	Reverse	CACGTGCCCTTCTATGAACC
COL4A1	Forward	CCTGGCTTGAAAAACAGCTC
	Reverse	CCCTGCTGAGGTCTGTGAAC
GGT1	Forward	GTGTTCTGCCGGGATAGAAA
	Reverse	CAGGTCCTCAGCTGTCACAA
GPC3	Forward	TGAAGATGAGTGCATTGGAGG
	Reverse	TGCTTATCTCGTTGTCCTTCG
HPRT1	Forward	TGACACTGGCAAAACAATGCA
	Reverse	GGTCCTTTTCACCAGCAAGCT
KRT19	Forward	CTCCGGGCACCGATCTCGC
	Reverse	AATTCTTCAGTCCGGCTGGTGAACC
LAMC1	Forward	AGATGGAAGCTGAGAATCTGG
	Reverse	CGGTCTGCTGTTCAGTCTTG
LGR5	Forward	GAAGCTCTGCAGAATTTGCGAAGCC
	Reverse	CAGGTGCCTCAGGGAATGCAGG
MYC	Forward	CTGGTGCTCCATGAGGAGA
	Reverse	CTCTGACCTTTTGCCAGGAG
RNA18S	Forward	CGCCGCTAGAGGTGAAATTC
	Reverse	TTGGCAAATGCTTTCGCTC
SNAI1	Forward	CCGACCCCAATCGGAAGCCTAAC
	Reverse	GGGGTTGAGGATCTCCGGAGGT
SOX9	Forward	TCTGGAGACTTCTGAACGAGAGCGA
	Reverse	CGTTCTTCACCGACTTCCTCCGC
TGFB1	Forward	AGTTGTGCGGCAGTGGTTGAGC
	Reverse	ACAGGAGCAGTGGGCGCTAAGG
TWIST	Forward	CATGTCCGCGTCCCACTAGCAG
	Reverse	CCCCACGCCCTGTTTCTTTGAATTTGG
NOTCH2	Forward	CAGTGTGCCACAGGTTTCACTG
	Reverse	GCATATACAGCGGAAACCATTCAC
HAPLN1	Forward	CACTGGACTTACAAGGTGTGG
	Reverse	TGCGCCTCGTGAAAATTGAG
TBP	Forward	GAGCTGTGATGTGAAGTTTCC
	Reverse	TCTGGGTTTGATCATTCTGTAG

Supplementary Table 1B:

TAQMAN PRIMERS & PROBES	
**GENE ID**	**Life Technologies cat. #**
RNA18S	Hs03003631-g1
HPRT1	Hs02800695-m1
LEF1	Hs01547250-m1
LGR5	Hs00173664-m1
NOG	Hs00271352-s1
IGFBP5	Hs00181213-m1
LFNG	Hs00385436-m1
HAPLN1	Hs00157103-m1
PI15	Hs00210658-m1

Supplementary Table 2: Primary antibodies.

PRIMARY ANTIBODIES		
**GENE ID**	**CAT. #**	**Supplier**
ACTA2	180106	Life Technologies
ALB	A80-229A	Bethyl Laboratories
PROM1	CD133/1 (AC133)	Miltenyi Biotech
CD44	20282	ProMab
CDH2	C3865	Sigma Aldrich
COL4A1	M0785	Dako
CTNNB1	610154	BD Biosciences
EPCAM	14-9326-82	eBioscience
FN1	F0916	Sigma Aldrich
GLUL	610518	BD Biosciences
GPC3	cmc26129021	Cell Marque
HAPLN1	HPA019105	Sigma Aldrich
HSC70	sc-7298	Santa Cruz
IGFBP5	I7660	Sigma Aldrich
KRT19	M0888	Dako
LFNG	AV44923	Sigma Aldrich
LGR5	HPA012530	Sigma Aldrich
SOX9	AB5535	Millipore
VIM	V6389	Sigma Aldrich

Supplementary Table 6: Wnt signals promote the differentiation of transit-amplifying liver progenitor cells to fibrogenic myofibroblast-like cells. As shown in the cartoon below, we compared two mRNA expression signatures:

(a) low density plating leading hepatocyte-like HepaRG cells to dedifferentiate to transit amplifying liver progenitor cells acquiring transient mesenchymal competence (GSE52989) [4] and

(b) Wnt3a leading transient mesenchymal competent cells to differentiate into myofibroblast-like cells.

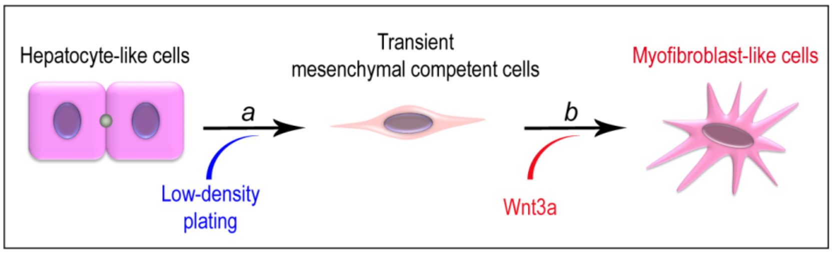

Supplementary Table 6A: Comparison of overlapping and non-overlapping genes between Wnt3a-induced transcriptome (signature ***b***), as described in Supplementary Table 3A, and the transient mesenchymal competent cell transcriptome (signature ***a***). Signature ***a***, obtained at 8; 12 and 16 hours after low-density plating of hepatocyte-like cells [4], was reanalyzed from the public dataset GSE52989, as follows:

$$Fold\ change = \frac{\left[\left(\frac{8\,h}{0\,h} + \frac{12\,h}{0\,h} + \frac{16\,h}{0\,h}\right)\right]}{3}$$

Eight, 12 and 16 hours (h) denote time after low-density plating (0 h).

Raw data were normalized by global scaling. The trimmed mean target intensity of each array was arbitrarily set to 500. Normalized and filtered entities were further filtered by gene symbol, retaining only those matching the 358-gene Wnt3a-induced

signature in HepaRG cells. Then, fold changes were calculated as the means of the 8 versus 0 h; 12 versus 0 h and 16 versus 0 h ratios as shown above.

The cells in the table below are colored according to the color code indicated at the bottom [red, >1.5 fold; orange, <1.5 and >0.7 fold (i.e. no change); green, <0.7 fold]. One hundred and seven genes which expression was differently modulated in both signatures are listed in the *"b"* column, with their corresponding fold changes. Ninety-three genes which were similarly regulated in both signatures are listed in the *"Common to a and b"* column, with their respective fold changes.

**Signature *b***	**Fold change in *b***	**Fold change in *a***	**Common to *a* and *b***	**Fold change in *b***	**Fold change in *a***
LRRC15	9.09	0.52	ACTN1	1.75	6.56
PI15	8.33	0.59	ADAM12	2.70	2.13
EPYC	4.17	0.50	ADRBK2	2.08	1.64
MMP13	3.85	1.40	ARHGDIB	1.72	48.39
COL1A1	3.70	0.57	ARL4C	2.44	1.73
KIAA1751	3.57	0.40	CCR1	1.52	1.58
FAP	3.57	1.28	CDCP1	1.61	17.53
MRC2	3.45	0.69	COL5A1	1.96	1.54
TCF7	3.33	0.83	CORO1C	1.49	9.10
PAMR1	3.23	0.40	CRISPLD2	2.08	1.55
ENO2	3.23	1.33	CYR61	1.82	1.64
CPEB1	3.03	1.08	DACT1	2.04	6.78
LHB	3.03	1.00	DCBLD2	1.75	3.40
CSF1R	2.94	0.33	DCN	1.72	3.60
LFNG	2.86	0.36	DPYSL3	1.59	2.69
PRR5L	2.86	0.56	ENDOD1	1.64	3.07
CSRP3	2.86	1.18	EPHB2	1.75	1.61
MMP7	2.86	1.01	EVI2A	3.03	3.53
MDFI	2.78	0.77	EXT1	1.67	1.84
SLC26A2	2.78	1.37	F3	2.17	28.90
VCAN	2.70	1.17	FHL2	1.56	10.95
CLIC3	2.63	0.40	FLNA	2.04	2.48
GNG4	2.56	1.12	GBP1	1.54	4.48
MMP2	2.56	0.76	GCNT1	2.56	3.45
PTPN22	2.50	1.13	GLIPR1	2.56	29.92
IHH	2.44	1.16	IGF2BP3	1.67	6.12

DLX5	2.38	0.25	ITGA5	1.61	3.09
PITX2	2.38	0.61	ITGB4	2.44	2.55
ADAM19	2.38	0.89	KLF7	1.61	2.36
SLC12A8	2.38	1.42	KLHL4	1.67	2.29
PDGFRL	2.33	0.66	LUM	2.08	3.29
IGFBP5	2.33	0.75	MKL1	1.61	2.59
KIF26B	2.27	0.29	MMD	1.89	3.21
FBN1	2.27	1.36	MYH9	1.67	2.91
LZTS1	2.27	0.80	MYL2	1.56	1.93
PKP3	2.27	1.24	PCSK5	5.26	3.40
SH3GL3	2.22	0.53	PDLIM3	1.96	1.51
OLFML3	2.22	0.75	PDLIM7	1.82	1.86
LEF1	2.17	1.39	PHLDB1	1.79	2.23
COL1A2	2.13	0.86	PLAUR	1.64	6.51
ARID3A	2.04	0.97	POTEKP	1.61	1.66
TEX15	2.04	0.72	PRSS23	1.85	5.76
NDRG4	2.00	0.59	PSTPIP2	1.61	3.08
LYPD1	2.00	1.28	RSU1	1.54	2.06
SLC7A5	1.96	0.61	SCG5	4.55	2.33
CLSTN2	1.96	0.73	SEMA3C	1.59	1.62
COL3A1	1.92	1.14	SERPINE1	1.72	18.51
CYP1B1	1.92	0.88	SH3BP4	1.56	2.17
CXCL3	1.89	0.63	SPOCK1	2.08	3.94
SLC38A3	1.89	0.49	SRPX2	1.69	3.88
SLC1A3	1.89	0.95	STK32B	1.89	1.59
MOXD1	1.85	0.94	TCOF1	1.56	1.81
TCHH	1.85	0.74	TGFB2	1.92	35.41
DNM3	1.82	0.35	TGFBI	2.17	2.09
FN1	1.82	0.51	THBS2	1.64	3.56
PCDH9	1.79	0.49	TMEM158	1.54	8.58
DPT	1.79	0.92	TNC	2.86	26.37
NXN	1.79	0.86	TNFAIP6	2.86	3.36
SPARC	1.79	1.38	TPM4	2.00	25.33
BMP1	1.75	0.99	ABCD2	0.41	0.26
EYA2	1.72	0.31	ACACB	0.54	0.16
P4HA2	1.72	0.87	ADARB2	0.45	0.36
RNF43	1.69	0.38	APOB	0.65	0.12
LCP1	1.69	0.89	BEST2	0.42	0.45
IL1R1	1.67	1.13	CYP4B1	0.24	0.19
ALDH7A1	1.64	0.56	CYP7B1	0.56	0.67
CSPG5	1.64	0.69	FA2H	0.41	0.33
RASSF9	1.64	0.56	FRAT1	0.60	0.30
BNC2	1.64	1.03	GCH1	0.58	0.62
ROS1	1.61	0.64	GHR	0.63	0.18
ARHGEF3	1.61	1.48	GPR116	0.59	0.50
NINJ2	1.61	0.79	HCG9	0.50	0.58
CCNG2	1.59	0.49	ITGA8	0.34	0.60

DISC1	1.59	0.42
JAM2	1.59	0.52
SDC3	1.59	1.30
STXBP6	1.59	0.71
CHPF2	1.56	1.26
DCAF15	1.56	0.88
FPR1	1.56	1.21
TYRP1	1.56	1.22
COL5A2	1.54	0.37
ALDH3A1	1.54	0.81
CFL1	1.54	1.18
JAG2	1.52	0.54
RARG	1.52	0.68
MDM1	1.52	0.76
ZFP2	0.67	0.90
DENND3	0.66	0.98
GRK4	0.65	0.92
PDHA1	0.65	0.91
TACC2	0.63	1.55
GPR98	0.62	0.81
DIO2	0.62	0.73
KRT4	0.62	0.87
RPGRIP1	0.61	1.13
DHRS9	0.60	16.77
MBP	0.59	1.15
HRASLS2	0.58	0.79
OCLN	0.57	0.82
FCGBP	0.56	0.70
JAKMIP2	0.54	2.59
PARD6B	0.53	7.59
SPINK5	0.52	1.19
AGR2	0.49	9.95
MARCO	0.48	1.81
S100P	0.44	0.76

KCNK5	0.53	0.18
LAIR1	0.62	0.34
LGALS2	0.44	0.17
LOC440792	0.50	0.68
LY6D	0.40	0.21
MB	0.60	0.63
MCF2	0.40	0.35
NR3C2	0.55	0.20
NRG2	0.60	0.34
PAIP2B	0.61	0.10
PDE8B	0.45	0.27
PRKAR2B	0.33	0.31
PSCA	0.29	0.58
RAB26	0.59	0.44
RALGPS1	0.61	0.38
RASGRP2	0.50	0.61
RASSF4	0.64	0.53
SLC6A16	0.64	0.56
SLIT2	0.53	0.41
SPTB	0.63	0.35

(red)	> 1.5 fold
(orange)	< 1.5; > 0.7 folds
(green)	< 0.7 fold

Supplementary Table 6B

Overrepresented gene ontology (GO) attributes in the 107 gene set induced by Wnt signals in HepaRG cells. Gene ontology enrichment analysis was performed with FuncAssociate 2.0 [1] using *hgnc_symbol* as namespace and $p<0.05$ as significance cutoff. *LOD* is the $\log_{10}$ of the odds value indicating overrepresentation.

Signature *(b):* Wnt-specific fibrogenic program

LOD	Adjusted *P*	Attribute ID	Overrepresented GO terms
2.02	0.002	GO:0005583	fibrillar collagen
1.52	<0.001	GO:0030199	collagen fibril organization
1.48	<0.001	GO:0071230	cellular response to amino acid stimulus
1.36	<0.001	GO:0030574	collagen catabolic process
1.32	<0.001	GO:0044243	multicellular organismal catabolic process
1.30	<0.001	GO:0032963	collagen metabolic process
1.27	<0.001	GO:0044259	multicellular organismal macromolecule metabolic process
1.26	<0.001	GO:0022617	extracellular matrix disassembly
1.24	<0.001	GO:0044236	multicellular organismal metabolic process
1.23	0.002	GO:0043200	response to amino acid
1.19	0.003	GO:0001649	osteoblast differentiation
1.07	0.002	GO:0001501	skeletal system development
1.03	<0.001	GO:0030198	extracellular matrix organization
1.03	<0.001	GO:0043062	extracellular structure organization
1.02	0.034	GO:0071229	cellular response to acid
1.01	0.002	GO:0044420	extracellular matrix part
0.99	<0.001	GO:0031012	extracellular matrix
0.88	0.022	GO:0005578	proteinaceous extracellular matrix
0.84	0.002	GO:0022411	cellular component disassembly
0.74	0.037	GO:0009887	organ morphogenesis
0.71	0.002	GO:0048731	system development
0.67	0.011	GO:0016477	cell migration
0.63	0.029	GO:0048870	cell motility
0.60	0.036	GO:0040011	locomotion
0.58	<0.001	GO:0032502	developmental process
0.58	<0.001	GO:0044767	single-organism developmental process
0.56	<0.001	GO:0048856	anatomical structure development
0.55	0.013	GO:0009653	anatomical structure morphogenesis
0.55	0.003	GO:0005576	extracellular region
0.54	0.036	GO:0005615	extracellular space
0.53	0.008	GO:0030154	cell differentiation
0.52	0.04	GO:0006928	cellular component movement

0.48	0.002	GO:0044699	single-organism process
0.46	0.018	GO:0048869	cellular developmental process
0.43	0.011	GO:0032501	multicellular organismal process
0.42	0.02	GO:0044707	single-multicellular organism process
0.40	0.011	GO:0044763	single-organism cellular process

Supplementary Table 6C

Common to signatures *(a)* and *(b)*: cell-extracellular matrix adhesion. (C) Overrepresented gene ontology attributes in the 93 gene set common to our Wnt3a and the Retrodifferentiation experiment by Dubois-Pot Scheneider *et al.* [4].

LOD	Adjusted *P*	Attribute ID	Overrepresented GO terms
2.43	0.002	GO:0051764	actin crosslink formation
1.04	0.001	GO:0005539	glycosaminoglycan binding
0.98	0.011	GO:1901681	sulfur compound binding
0.92	<0.001	GO:0030198	extracellular matrix organization
0.92	<0.001	GO:0043062	extracellular structure organization
0.86	0.025	GO:0031012	extracellular matrix
0.84	0.004	GO:0007411	axon guidance
0.84	0.004	GO:0097485	neuron projection guidance
0.74	<0.001	GO:0007155	cell adhesion
0.74	<0.001	GO:0022610	biological adhesion
0.65	0.031	GO:0005856	cytoskeleton
0.61	0.001	GO:0006928	cellular component movement
0.58	0.007	GO:0009653	anatomical structure morphogenesis
0.54	0.016	GO:0005576	extracellular region
0.49	<0.001	GO:0032502	developmental process
0.48	<0.001	GO:0044767	single-organism developmental process

Supplementary Table 7: Gene expression correlations between the indicated genes in 79 human HCCs. Pearson's correlation coefficients ≥0.30 were integrated in expression networks with Cytoscape and are shown in Fig. 3E.

	LGR5	PI15	HAPLN1	IHH	LFNG	NOG	IGFBP5	LEF1	COL4A1	LAMC1	ACTA2	SFRP1	SFRP2	DKK1	FZD7	FZD1	WNT2	WNT3	SOX9	GPC3	CD44	KRT19
**PI15**	0.39																					
**HAPLN1**	0.23	0.26																				
**IHH**	0.25	0.26	0.21																			
**LFNG**	0.04	0.28	0.27	0.18																		
**NOG**	-0.14	0.08	0.04	0.64	0.15																	
**IGFBP5**	0.22	0.37	0.34	0.15	0.73	0.14																
**LEF1**	0.51	0.50	0.32	0.23	0.58	0.07	0.57															
**COL4A1**	0.00	0.04	0.37	0.00	0.29	0.08	0.28	0.17														
**LAMC1**	0.10	0.28	0.35	0.18	0.20	0.04	0.22	0.34	0.46													
**ACTA2**	-0.06	0.21	0.06	0.06	0.27	0.08	0.25	0.20	0.50	0.56												
**SFRP1**	-0.11	0.09	0.02	-0.19	0.34	-0.10	0.24	0.07	0.46	0.35	0.36											
**SFRP2**	-0.14	0.02	0.08	0.07	0.22	0.26	0.23	0.16	0.41	0.30	0.40	0.47										
**DKK1**	-0.03	0.01	-0.03	0.07	-0.19	0.04	-0.09	-0.02	0.17	0.34	0.21	0.13	0.43									
**FZD7**	-0.10	0.09	0.11	0.10	0.13	0.14	0.20	0.00	0.23	0.39	0.17	0.37	0.31	0.30								
**FZD1**	0.08	-0.03	0.13	0.37	-0.05	0.34	-0.06	0.06	0.07	0.11	0.00	-0.06	0.38	0.40	0.16							
**WNT2**	-0.04	0.10	0.15	0.18	-0.10	0.22	-0.07	-0.02	0.34	0.35	0.37	0.22	0.47	0.49	0.40	0.39						
**WNT3**	0.09	-0.16	-0.12	-0.10	-0.29	-0.09	-0.16	-0.11	-0.20	-0.11	-0.14	-0.02	0.13	0.07	-0.02	0.14	-0.07					
**SOX9**	-0.08	0.03	0.07	-0.04	0.42	0.13	0.45	0.16	0.17	-0.08	0.08	0.22	0.29	-0.01	0.23	0.03	-0.04	-0.16				
**GPC3**	0.35	0.29	0.14	0.32	0.23	0.04	0.28	0.46	-0.12	-0.05	-0.02	-0.11	-0.11	-0.06	-0.09	0.01	-0.20	-0.05	0.11			
**CD44**	0.23	0.37	0.18	-0.06	0.57	-0.14	0.44	0.53	0.05	0.05	0.14	0.25	0.13	-0.20	0.01	-0.23	-0.14	-0.03	0.36	0.39		
**KRT19**	-0.16	0.15	0.17	0.08	0.30	0.15	0.31	0.15	0.13	0.00	0.18	-0.03	0.22	-0.16	-0.02	0.13	0.09	-0.02	0.23	-0.11	0.14	
**EPCAM**	-0.16	0.14	0.08	0.10	0.29	0.18	0.26	0.13	0.05	-0.02	0.18	0.00	0.24	-0.07	-0.01	0.10	0.02	0.00	0.22	-0.21	0.11	0.87

Supplementary clinical observations:

AFP levels above the median (6.3 IU/ml) are associated with bad outcome. Platelet counts above the median ($147x10^3$/µl) protected from early death. As thrombocytopenia is a common complication in cirrhosis [5] and 90% of HCCs in this cohort arose in cirrhotic livers (Supplementary Table 8), this finding seems related to the underlying liver disease. Consistently with a recent study [6], dyslipidemia protected from early death.

Supplementary Table 10: Search for CTNNB1 exon 3 mutations in 82 samples of human HCC by Sanger sequencing.

Sample ID	CTNNB1 Exon3
1	WT
2	WT
3	c.A121C/p.T41P
4	c.C134T/p.S45F
5	c.A121C/p.T41P
6	WT
7	c.del107-109 p.H36-S37>P
8	c.A121C/p.T41P
9	WT
10	WT
11	c.A95T/p.D32V
12	WT
13	c.C134T/p.S45F
14	c.A107C/p.H36P
15	c.C134T/p.S45F
16	WT
17	WT
18	WT
19	WT
20	WT
21	WT
22	WT
23	c.G94T/p.D32Y
24	c.A107C/p.H36P
25	WT
26	WT
27	WT
28	WT
29	WT
30	WT
31	c.G94T/p.D32Y
32	WT
33	WT
34	WT
35	WT
36	WT
37	c.A107C/p.H36P
38	WT
39	c.C122T/p.T41I
40	WT
41	WT
42	WT
43	c.A121C/p.T41P
44	WT
45	WT
46	WT
47	c.C122T/p.T41I
48	WT
49	WT
50	WT
51	WT
52	WT
53	c.G101T/p.G34V
54	WT
55	WT
56	WT
57	WT
58	WT
59	WT
60	WT
61	WT
62	WT
63	WT
64	c.C134A/p.S45Y
65	WT
66	WT
67	c.A95T/p.D32V
68	c.A95G/p.D32G
69	WT
70	c.G101T/p.G34V
71	c.A121G/p.T41A
72	WT
73	WT
74	WT
75	c.A95G/p.D32G
76	WT
77	WT
78	c.C110G/p.S37C
79	WT
80	WT
81	WT
82	c.G94T/p.D32Y

References to supplementary data

1. Berriz GF, Beaver JE, Cenik C, Tasan M and Roth FP. Next generation software for functional trend analysis. Bioinformatics. 2009; 25(22):3043-3044.

2. van Zijl F, Mall S, Machat G, Pirker C, Zeillinger R, Weinhaeusel A, Bilban M, Berger W and Mikulits W. A human model of epithelial to mesenchymal transition to monitor drug efficacy in hepatocellular carcinoma progression. Molecular cancer therapeutics. 2011; 10(5):850-860.

3. Supek F, Bosnjak M, Skunca N and Smuc T. REVIGO summarizes and visualizes long lists of gene ontology terms. PloS one. 2011; 6(7):e21800.

4. Dubois-Pot-Schneider H, Fekir K, Coulouarn C, Glaise D, Aninat C, Jarnouen K, Le Guevel R, Kubo T, Ishida S, Morel F and Corlu A. Inflammatory cytokines promote the retrodifferentiation of tumor-derived hepatocyte-like cells to progenitor cells. Hepatology. 2014; 60(6):2077-2090.

5. Hayashi H, Beppu T, Shirabe K, Maehara Y and Baba H. Management of thrombocytopenia due to liver cirrhosis: a review. World journal of gastroenterology : WJG. 2014; 20(10):2595-2605.

6. Chiang CH, Lee LT, Hung SH, Lin WY, Hung HF, Yang WS, Sung PK and Huang KC. Opposite association between diabetes, dyslipidemia, and hepatocellular carcinoma mortality in the middle-aged and elderly. Hepatology. 2014; 59(6):2207-2215.
